# A Study Based on Network Pharmacology Decoding the Multi-Target Mechanism of Duhuo Jisheng Decoction for the Treatment of Intervertebral Disc Degeneration

**DOI:** 10.1155/2023/7091407

**Published:** 2023-05-28

**Authors:** Hao Liu, Yumin Li, Zhujun Li, Jie Li, Qiongchi Zhang, Shuai Cao, Haopeng Li

**Affiliations:** ^1^Department of Orthopedics, The Second Affiliated Hospital of Xi'an Jiaotong University, Xi'an, China; ^2^Department of Orthopedics, Civil Aviation General Hospital, No. 1, Gaojing Street, Chaoyang District, Beijing 100123, China

## Abstract

Intervertebral disc degeneration (IDD) poses a grim public health impact. Duhuo Jisheng Decoction (DJD), a traditional Chinese medicine formula, has recently received significant attention for its efficacy and safety in treating IDD. However, the pathological processes of IDD in which DJD interferes and molecular mechanism involved are poorly understood, which brings difficulties to the clinical practice of DJD for the treatment of IDD. This study systematically investigated the underlying mechanism of DJD treatment of IDD. Network pharmacology approaches were employed, integrating molecular docking and random walk with restart (RWR) algorithm, to identify key compounds and targets for DJD in the treatment of IDD. Bioinformatics approaches were used to further explore the biological insights in DJD treatment of IDD. The analysis identifies AKT1, PIK3R1, CHUK, ALB, TP53, MYC, NR3C1, IL1B, ERBB2, CAV1, CTNNB1, AR, IGF2, and ESR1 as key targets. Responses to mechanical stress, oxidative stress, cellular inflammatory responses, autophagy, and apoptosis are identified as the critical biological processes involved in DJD treatment of IDD. The regulation of DJD targets in extracellular matrix components, ion channel regulation, transcriptional regulation, synthesis and metabolic regulation of reactive oxygen products in the respiratory chain and mitochondria, fatty acid oxidation, the metabolism of Arachidonic acid, and regulation of Rho and Ras protein activation are found to be potential mechanisms in disc tissue response to mechanical stress and oxidative stress. MAPK, PI3K/AKT, and NF-*κ*B signaling pathways are identified as vital signaling pathways for DJD to treat IDD. Quercetin and Kaempferol are assigned a central position in the treatment of IDD. This study contributes to a more comprehensive understanding of the mechanism of DJD in treating IDD. It provides a reference for applying natural products to delay the pathological process of IDD.

## 1. Introduction

Low back pain (LBP) is a significant cause of chronic pain worldwide. Intervertebral disc degeneration (IDD), considered to be the leading cause of LBP, is the pathological basis of multiple disc degenerative diseases (DDD) such as intervertebral disc herniation and spinal stenosis, which poses a massive burden to health and the economy [1, 2]. If conservative treatment fails, spinal fusion is considered the current “gold standard” for DDD [3]. Although significant progress such as gene therapy, stem cell therapy, and bioengineering treatment was developed in the treatment of IDD, the potency of these new technologies is limited by the unique anatomical features of the intervertebral disc, the harsh microenvironment of the degenerative intervertebral disc (such as high glucose and oxidative stress), and the technical limitations of the technology itself [4–6]. More exploration is needed before the new technology can be translated into practical clinical applications, at which time drugs play an irreplaceable role in the treatment of IDD [6–8].

Natural products have often been used in Chinese medicine for centuries to treat many diseases. Duhuo Jisheng Decoction (DJD), a traditional Chinese formula, was considered to have the functions of nourishing the liver and kidney, activating qi, and promoting blood circulation according to traditional Chinese medicine theory [9]. It has been used to treat osteoarthritis in the past due to its anti-autophagy and anti-inflammatory effects [10]. Recently, several systematic reviews have stated the safety and efficacy of DJD in slowing the progression of IDD and alleviating LBP, demonstrating the value of DJD in the treatment of IDD [11, 12]. A study confirmed in human degenerative nucleus pulposus cells (NPCs) in vitro that DJD inhibited the inflammation of NPCs and the reduction of extracellular matrix. Also, this way of inhibiting the inflammatory response may be by inhibiting the NF-*κ*B pathway [13]. Furthermore, another study using compression-induced aging of the intervertebral disc in a rat model found that DJD can activate autophagy and significantly reduce apoptosis of NPCs and matrix degeneration. Further research found that DJD may lead to the corresponding biological behavior of NPCs by inhibiting the MAPK pathway [14]. Previous studies have reported that the MAPK pathway and NF-*κ*B pathway may play a role in the treatment of IDD by DJD [13, 14], but the molecular mechanism by which DJD targets the above pathways is unclear. Moreover, there are many bioactive compounds in DJD, which may involve more pathological processes and molecular mechanism, but there is currently a lack of systematic understanding of the mechanism. Network pharmacology mining the associations between drug and disease targets is a novel and promising strategy to reveal the complex mechanism of disease and identify new therapeutics [10]. Molecular docking is an important technique in the field of computer-aided drug research, which is used to predict the affinity and binding properties of drugs to specific targets, and has become a mature technology in pharmacological research [15]. The present study employed network pharmacology and molecular docking techniques to investigate the specific molecular mechanism of DJD regulating MAPK pathway and NF-*κ*B pathway to treat IDD. It systematically explored the underlying mechanisms of DJD in the treatment of IDD, aiming to enhance a more comprehensive understanding of the mechanism of DJD in the treatment of IDD and provide valuable insights for the application of natural products in delaying IDD.

## 2. Methods

### 2.1. DJD Target Screening and Toxicity Prediction

The Traditional Chinese Medicine Systems Pharmacology Database and Analysis Platform (TCMSP, https://tcmspw.com/tcmsp.php) was adapted to search the compounds of DJD and obtain the structural parameters of these compounds. Based on the structural parameters, Lipinski's rule of five [16] (RO5, details are provided in Table S1) and oral bioavailability (OB) were used to construct a filter to assess the oral potential of compounds: OB > 30% or meet the conditions of RO5. Moreover, other three parameters which were previously recommended were employed to screen out the compounds with higher bioactivity and drug-likeness: Caco-2 permeability > 0.4, drug-like (DL) properties > 0.18, and half-life > three hours [15, 17]. DrugBank (https://go.drugbank.com/), Swiss Target Prediction (https://www.swisstargetprediction.ch/), and TargetNet web server (https://targetnet.scbdd.com) were adapted to collect the targets of the qualified compounds, and only targets with a probability degree greater than 0.8 were included [18]. The Protox II web server (https://tox-new.charite.de/protox_II/) was then used to make toxicity predictions for these qualified compounds [19].

### 2.2. Collection of Targets Related to IDD

We collected IDD-related targets from DisGeNET (https://www.disgenet.org/) and GeneCards (https://www.genecards.org/) databases [20]. It should be noted that GeneCards employs a parameter “Score” to indicate the relevance of the retrieval results to the subject terms used for search. In order to include targets that are more relevant to IDD, we sorted the targets retrieved on GeneCards in descending order of Score and only included the top 50 percent of the targets. In addition, we also collected genes enriched in MAPK, P38-MAPK, PI3K/AKT, Wnt/*β*-catenin, ATM-p53-P21-Rb, and mTOR signaling pathways from PathCards (https://pathcards.genecards.org/) [16]. Since the pathways mentioned above are reported to be closely related to IDD [21–23], the genes in these pathways and IDD-related targets were intersected and labeled as potentially important targets.

### 2.3. Compound-Target Network Construction and Analysis

We used the Venn method to obtain overlapping targets of DJD and IDD as common targets for drugs and diseases. However, a single compound may be shared by multiple botanical drugs, which complicates understanding the relationship among the botanical drugs, compounds, and targets. To show this complicated relationship more intuitively, we renamed the compounds contained in the various botanical drugs in DJD according to the following rules: the compounds numbered “A” to “H” represented compounds shared by two or more botanical drugs; the name of a compound consisting of an abbreviation plus an Arabic numeral suffix indicates that the herb represented by the abbreviation uniquely occupies the compound. The renaming results of compounds in DJD are provided in Table S2. Then, a compounds-targets network was generated using Cytoscape (v3.8.2). The degree of nodes representing each compound in the network was analyzed using CytoNCA (a Cytoscape plugin). We then analyzed the protein functions encoded by these common target genes and the network of transcription factors that regulate them. The above information was retrieved through the Panther classification system (https://pantherdb.org/) and Transcriptional Regulatory Relationships Unraveled by Sentence-based Text mining (TRRUST, https://www.grnpedia.org/trrust/), respectively [24]. A bar graph summarizing the transcriptional regulatory network of common target genes was generated from Metascape (https://metascape.org/gp/index.html/).

### 2.4. Construction of the Protein-Protein Interaction (PPI) Network of Common Targets and Identification of Key Targets and Protein Complexes or Functional Modules

The common targets obtained through the Venn method were uploaded to the STRING database (https://string-db.org/) with the species limited to “9606” (human), to collect protein-protein interaction (PPI) information [16]. We then screened for interactions with confidence scores greater than 0.4 and built the PPI network on Cytoscape (v3.8.2). The topological analysis of the resulting PPI network was performed using CytoNCA (a Cytoscape plugin). The parameters obtained from the analysis were imported into R software 4.1.2 to screen the nodes with degree and betweenness greater than 2 times the median in the PPI network as the core targets. In addition, in living phenomena, proteins usually form complexes or functional modules to function. So, we further use the “Molecular Complex Detection” (MCODE) algorithm to investigate the underlying protein complexes or functional modules in the PPI network [25].

### 2.5. Analysis of Biological Insights

Gene Ontology (GO) and Kyoto Encyclopedia of Genes and Genomes (KEGG) pathway analyses were conducted by R package “clusterProfiler” on these common targets, *p* value <0.005 was used as the cutoff to be considered significantly enriched, and R package “ggplot2” was used to visualize the enrichment results [26]. The restart random walk algorithm with restart was employed to evaluate the influence of DJD targets on a PPI network to screen genes with specific biological significance [27]. Then, biological function analysis of genes based on co-expression correlations was performed using the R package “Correlation AnalyzeR” [28], defining a predesigned gene correlation matrix as a “cartilaginous” tissue source. Two similar groups in the PPI network can exhibit similar biological effects. Here we used the proximity index proposed by Menche et al. [29] to calculate the proximity index to understand the role of DJD in the biological process of interest. The calculation formula of the network proximity index of group A genes and group B genes is as follows:

(1) $$\begin{array}{c} s_{AB}=\langle d_{AB}\rangle -\frac{\langle d_{AA}\rangle -\langle d_{BB}\rangle }{2}\text{.} \end{array}$$

The value sAB < 0 indicates that the targets of the two groups are located in the same neighborhood, suggesting similar effects. We collected genes related to cellular inflammatory response, autophagy, and apoptosis on GeneCards. We then screened the same number of genes as DJD targets from the above three gene sets based on higher correlation with biological processes and calculated their network proximity index to DJD targets. Then, we randomly selected 50 groups of genes with the same number of DJD targets from the human protein interaction network containing 10,995 genes constructed on STRING and calculated the average value of their network proximity index to DJD targets as control.

### 2.6. Molecular Docking

Before carrying out molecular docking, we identified binding site of each receptor protein by reviewing the literature as well as referring to the co-ligand binding site of the receptor protein. For receptor proteins lacking evidence to identify binding sites, POCASA (https://altair.sci.hokudai.ac.jp/g6/service/pocasa/) [30] was used to predict their active pockets as binding sites. We then downloaded the 3D structure of the receptor proteins containing the binding sites from the RCSB-PDB database (https://www.rcsb.org/) [15]. The SMILE format files of the small molecule ligands were obtained from PubChem (https://pubchem.ncbi.nlm.nih.gov/) [27]. UCSF Chimera (1.16) was used to generate 3D structures through the SMILE format of small molecule ligands [31]. Information on the receptor proteins and small molecule ligands employed by our docking is provided in Table S3. Then, we used UCSF Chimera (1.16) to optimize receptor proteins and small molecule ligands (minimization routines are provided by MMTK, which is included with Chimera) [31]. We then imported the optimized receptor proteins and small molecule ligands into AutoDockTools for predocking preparation. We performed removal of water molecules, addition of hydrogen atoms, calculation of charge, and addition of atom type to the receptor proteins. Operations performed on small molecule ligands include adding hydrogen atoms, adjusting charges, determining roots, and detecting and setting torsional bonds. After completing the predocking preparation, we set the parameters of the docking box according to the binding sites identified previously. The parameters of the docking box are recorded in Table S3. We then performed molecular docking using AutoDock Vina with docking set to be based on AutoDock4 force field and exhaustiveness set to 32 (when the exhaustiveness is greater than 25, more resource consumption can only bring little benefit to the scoring function; we set exhaustiveness to 32 to ensure accuracy when performing molecular docking) [32]. After molecular docking, the PLIP web tool (https://plip-tool.biotec.tu-dresden.de/plip-web/plip/index) was used to analyze the protein-ligand interaction [33]. UCSF Chimera (1.16) was used to visualize the docking results [31].

## 3. Results

### 3.1. Compounds of DJD and Common Targets

**Figure 1:**
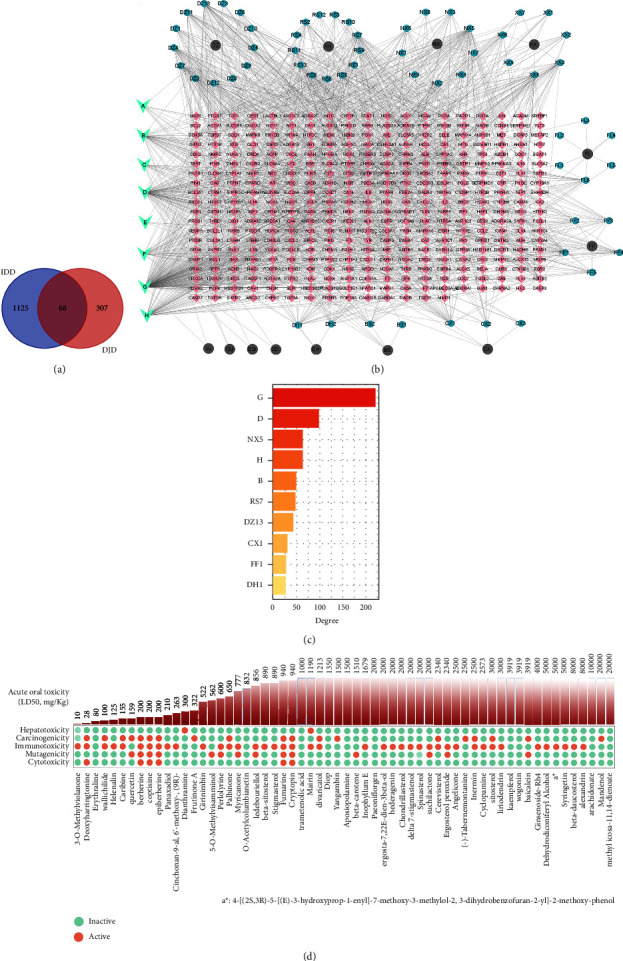
Compound-target network and compound toxicity parameter prediction. (a) Venn diagram of the common targets of DJD and IDD. (b) Network of compounds of DJD and common targets. Each pink rectangular node represents a common target, and each gray dot represents a herb. The nodes in the circles around these gray dots represent the compounds contained in the botanical drugs. The nodes marked “*A*” to “*H*” represent the compounds in DJD that are shared by two or more botanical drugs. The specific meaning of a single node is represented by label text in the node. (c) The top ten compounds of degree in the compound-target network. (d) Predicted toxicity parameters of screened compounds in DJD.

67 compounds of DJD are screened out by employing the filter, ensuring the compounds have oral bioavailability and therapeutic potential (Table S4). The toxicity parameters of these compounds were predicted through the Protox II web server to assess their toxicity.  Figure 1(a) describes the potential of these compounds in terms of hepatotoxicity, carcinogenicity, immunotoxicity, mutagenicity, cytotoxicity, and acute oral toxicity (LD50, mg/kg). As shown in Figure 1(d), only 2 compounds (Dianthramine and Mairin) are predicted to be hepatotoxic. In addition, the acute oral toxicity of Dianthramine and Mairin is predicted to be at a high level of 300 mg/kg and 1190 mg/kg, respectively. The above results suggest that DJD has a comparatively lower risk of hepatotoxicity. However, the toxicity risks of DJD in terms of cytotoxicity, immunotoxicity, and cardiotoxicity need to be considered. Many of the compounds are predicted to exhibit cytotoxicity, immunotoxicity, and cardiotoxicity, and the acute oral toxicity of some compounds is at a low level (such as Deoxyharringtonine, 3-O-Methylviolanone, and Wallichilide). Although none of the compounds with mutagenic toxicity in DJD are at the low level of acute oral toxicity, there are still many compounds predicted to have mutagenic toxicity. Further evaluation of the risk of mutagenic toxicity of DJD is needed. The above provides information for balancing the efficacy and safety of DJD. Then, according to the constraints in the method to ensure high confidence of targets, a total of 375 DJD-related targets and 1193 IDD-related targets are finally included. 68 common targets of DJD and IDD are identified through the Venn method (Figure 1(a)).

### 3.2. Construction of a Compound-Target Network of DJD and Gaining Insight into Key Compounds from a Network Perspective

A network of botanical drugs, compounds, and common targets was constructed to help understand the complicated interactions between them (Figure 1(b)). Compounds represented by the letters “*A*” through “*H*” indicate their presence in two or more botanical drugs of DJD. They are Mairin, *β*-Sitosterol, Sitosterol, Kaempferol, Mannitol, Stigmasterol, Quercetin, and Wogonin, respectively. Among them, Beta-Sitosterol, Sitosterol, and Kaempferol are the three most widely distributed compounds, which exist in 8, 6, and 5 botanical drugs, respectively (Table S2). The abovementioned compounds are widely distributed in botanical drugs, suggesting that they are an important part of the DJD compound library, which to some extent reflects that they may play an important role in the treatment of DJD. More importantly, Figure 1(c) indicates that Quercetin (G), Kaempferol (D), Baicalein (NX5), Wogonin (H), Beta-Sitosterol (B), Frutinone A (RS7), Syringetin (DZ13), 5-O-Methylvisamminol (CX1), Myricanone (FF1), and Angelicone (DH1) are the compounds with the top 10 degrees in the network, suggesting that they form denser connections with common targets and thus may play an important role in the treatment of IDD by DJD. It is worth noting that the degrees of Quercetin and Kaempferol are 212 and 102, respectively, which are significantly higher than those of other compounds in the network. The above results indicate that Quercetin and Kaempferol have greater potential for multi-target intervention than other compounds and are the key compounds of DJD in the treatment of IDD.

### 3.3. Protein Function Classification and Transcriptional Regulatory Network

**Figure 2:**
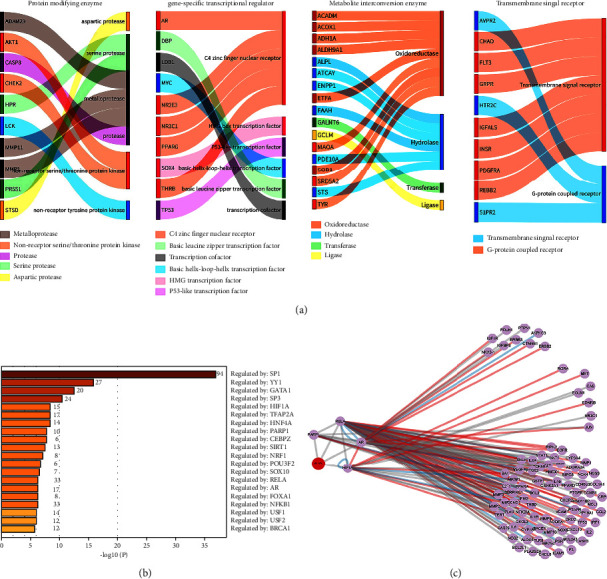
Analysis of protein function classification and transcriptional regulatory factor of common target genes. (a) Sankey diagram of the main four protein functional classes of common target genes. (b) Bar graph summary of enrichment analysis in TRRUST. The *p* value was calculated based on the cumulative hypergeometric distribution, and “Log10 (*p*)” was the log-base*p* value of 10. The label number represents the count of genes regulated by that gene. (c) Transcriptional regulatory networks of transcriptional regulators in DJD. The connection between nodes: red for “activation,” blue for “repression,” and gray for “unknown.”

Function classification of the proteins encoded by these common targets reveals that metabolite interconversion enzyme, transmembrane signal receptor, protein-modifying enzyme, and gene-specific transcriptional regulator are the most distributed groups, with 17, 10, 10, and 10 target enrichment (Figure 2(a)). Interestingly, oxidoreductase is the primary type in metabolite interconversion enzymes, including SOD1, ACADM, ACOX1, ADHA1, ALDH9A1, ETFA, MAOA, SRD5A2, and TYR, suggesting that DJD has the potential to regulate cellular oxidative stress (Figure 2(a)). Oxidative stress is considered to be one of the initial factors inducing nucleus pulposus cell senescence, so DJD may regulate oxidative stress as one of the mechanisms of its treatment of IDD [5]. Protein-modifying enzymes include AKT1, ADAM29, CASP8, CHEK2, HPR, LCK, MMP11, MMP1, PRSS1, and STSD (Figure 2(a)). AKT1, as a non-receptor serine protein kinase, regulates the conversion of activated forms of proteins and is essential for signal transduction. AKT1 plays a vital role in various signaling pathways such as the MAPK and PI3K/AKT pathways and also plays an essential role in gene transcription mediated by NF-*κ*B pathway [34, 35]. Moreover, these pathways were previously reported to be related to IDD [13, 14, 21, 22]; therefore, DJD may target AKT1 to regulate the above pathways and thus treat IDD. MMP1 and MMP11 are members of matrix metalloproteinases (MMPs), which are key enzymes in the degradation of extracellular matrix and affect the balance of synthesis and catabolism of extracellular matrix in nucleus pulposus [36]. The imbalance of extracellular matrix metabolism directly leads to the morphological changes of intervertebral disc and accelerates the degeneration process [37]. Therefore, the effect of DJD on extracellular matrix metabolism by targeting MMPs may serve as a potential mechanism for its treatment of IDD. As for the cell surface transmembrane signal receptors binding ligands, DJD includes transmembrane signal receptor and G-protein coupled receptor, and the former is the primary (Figure 2(a)). C4 zinc finger nuclear receptors are major gene-specific transcriptional regulators in DJD, including AR, NR2E3, NR3C1, PPARG, and THRB (Figure 2(a)). The transcriptional regulatory network of DJD target genes was analyzed.  Figure 2(b) shows the number of genes regulated by all genes playing transcriptional regulatory roles and their enrichment ranking in the network. SP1, as a core member of the transcriptional regulatory network, regulates the transcription of 94 target genes, followed by YY1, which regulates 27 genes, and GATA1, which regulates 20 target genes. It is worth noting that HIF1A, PARP1, RELA, and AR are not only transcriptional regulators but also target genes of DJD.  Figure 2(c) shows the regulatory relationship between the above four transcriptional regulators and target genes of DJD. Among them, RELA has an extensive regulatory relationship with other target genes, which suggests that it is an essential transcriptional regulator for DJD treatment of IDD.  Table 1 details the biological processes associated with IDD regulated by these transcription factors, mainly involving the regulation of cellular senescence, apoptosis, inflammation, mechanical stress, and hypoxia. Mechanical stress, inflammation, and hypoxia affect the senescence and apoptosis of NPCs, which are closely related to IDD [38]. These results suggest that DJD may regulate specific transcription factors to affect degeneration of disc at the transcriptional level.

### 3.4. Identification of Key Targets and Results of Molecular Docking

**Figure 3:**
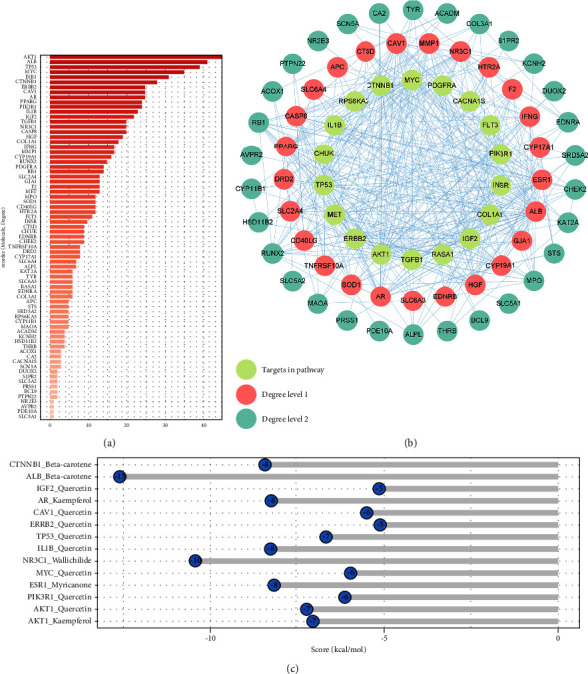
The PPI network of common target genes and result of molecular docking. (a) The degree value of each gene in the PPI network of common target genes. (b) The PPI network of common target genes. The degree level is divided by the median of the degree value as the cutoff point. (c) Molecular docking results. Scores below −5 kcal/mol indicate stable docking.

**Figure 4:**
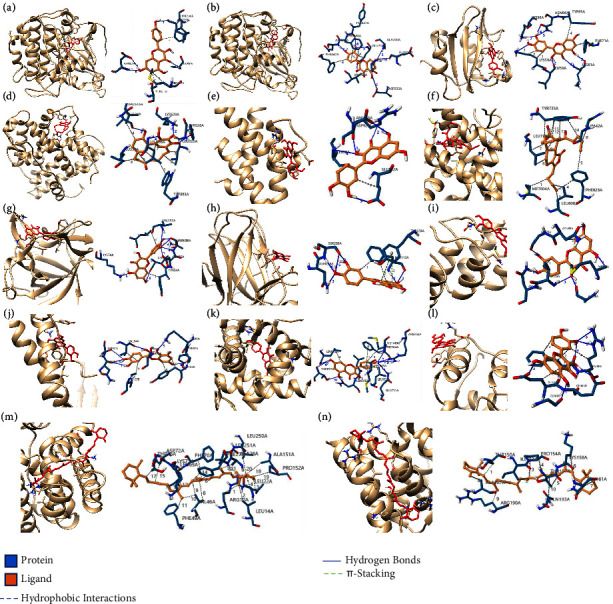
Molecular docking models. (a) The binding of Kaempferol to AKT1. (b) The binding of Quercetin to AKT1. (c) The binding of Quercetin and PIK3R1. (d) The binding of Myricanone and ESR1. (e) The binding of Quercetin and MYC. (f) The binding of Wallichilide and NR3C1. (g) The binding of Quercetin and IL1B. (h) The binding of Quercetin and TP53. (i) The binding of Quercetin and ERBB2. (j) The binding of Quercetin and CAV1. (k) The binding of Kaempferol and AR. (l) The binding of Quercetin and IGF2. (m) The binding of Beta-Carotene and ALB. (n) The binding of Beta-Carotene and CTNNB1. Arabic numerals represent the index, mapping the interactions in Table S5.

A PPI network was constructed with 68 common targets (Figure 3(b)). The PPI network has 402 edges, the average node degree is 11.8, and the average local clustering coefficient is 0.666. Among the common targets, CTNNB1, MYC, PDGFRA, CACNA1S, FLT3, PI3KR1, INSR, COL1A1, IGF2, RASA1, TGFB1, AKT1, ERBB2, MET, TP53, CHUK, IL1B, and RPS6KA3 are marked as IDD-related pathway members (Figure 3(b)), 15 in MAPK pathway, 13 in PI3K/AKT pathway, 2 in Wnt/*β*-catenin pathway, and 1 in ATM-p53-P21-Rb pathway. Most of them were of high degree (Figure 3(a)) and interacted extensively with other common targets in the PPI network, suggesting that those targets may play an important role in the treatment of IDD by DJD. The network topology analysis identified 13 key targets, including AKT1, PIK3R1, ALB, TP53, MYC, NR3C1, IL1B, ERBB2, CAV1, CTNNB1, AR, IGF2, and ESR1. These key targets are at the core of this PPI network and closely interact with other common targets, suggesting their central role in DJD treatment of IDD, especially AKT1, as it exhibits the highest degree (degree = 48, Figure 3(a)). The results of molecular docking show that the binding free energy ranged from −5.12 to −12.61 kcal/mol and the inhibition constant (Ki) ranged from 0.57 × 10^−3^ to 175.26 *μ*mol/ml (Figure 3(c), Table 2).  Figure 4 visualizes the binding of receptor proteins and their small molecule ligands. The results of protein-ligand interaction are shown in Table S5. The protein-ligand interaction analysis shows that there are mainly hydrophobic interactions and hydrogen bonds between receptor proteins and small molecule ligands. In addition, there is a *π*-stacking interaction between TP53 and Quercetin. The above results suggest that the core targets as receptor proteins can form solid binding with the corresponding small molecule ligands in DJD. Moreover, the MCODE algorithm investigated the PPI network's protein complexes or functional modules. The analysis results show that one module is detected, while all the key targets are distributed in this module (Figure S1), further indicating that these key targets play an essential role in the treatment of IDD by DJD.

### 3.5. DJD Regulates Cellular Mechanical Stress Response and Reactive Oxygen Species Processing

**Figure 5:**
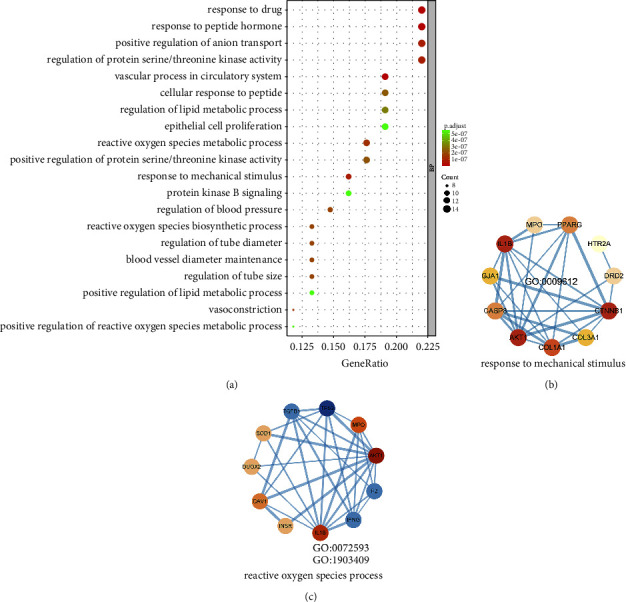
GO analysis of the common targets. (a) The top 20 BP enrichment analysis results. (b) The PPI network of common target genes that were enriched in response to mechanical stimulus (GO: 0009612). (c) The PPI network of common target genes enriched in reactive oxygen species metabolic process (GO: 0072593) and reactive oxygen species biosynthetic process (GO: 1903409).

**Figure 6:**
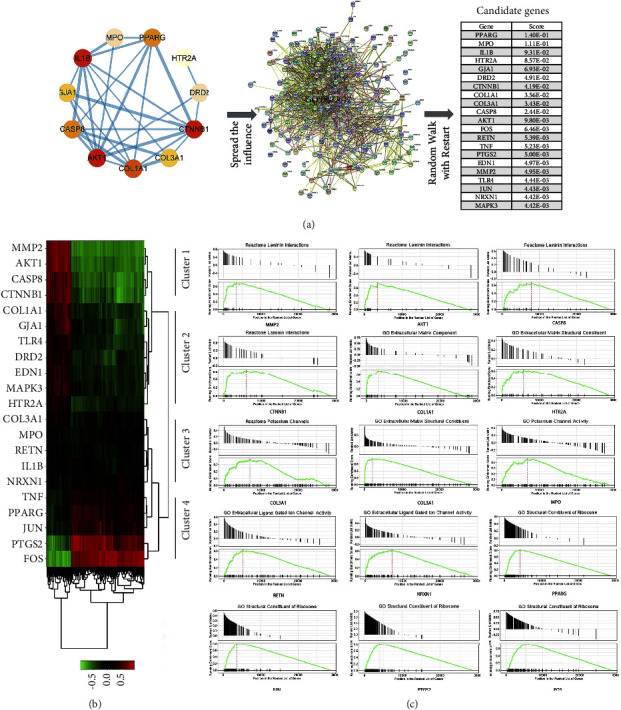
DJD modulates the response to mechanical stimulus in the intervertebral disc. (a) The network spread score of gene set related to mechanical stress response in common target genes in the PPI network of response to mechanical stimulation (GO: 0009612) was calculated using the random walk with restart algorithm, and top 10 were selected as candidate genes for subsequent analysis. (b) In the candidate gene set, gene groups were identified by the “Correlation AnalyzeR” R package as the members of the set who share correlations in common that are not shared with other members, thereby classifying genes with common biological functions. (c) The correlation-based gene set enrichment analysis results of genes in each cluster.

**Figure 7:**
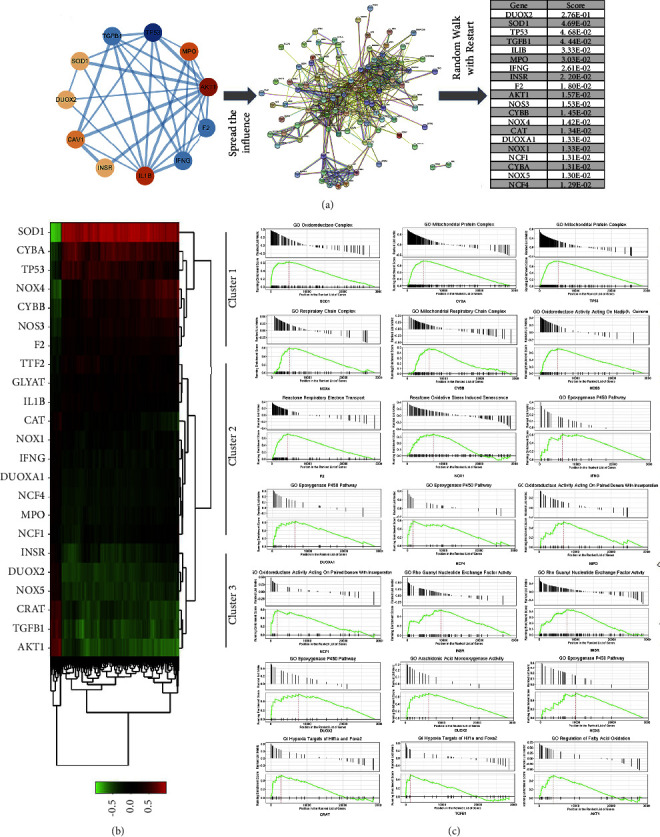
DJD modulates the response to mechanical stimulus in the intervertebral disc. (a) The network spread score of gene set related to mechanical stimulation in common target genes in the PPI network of response to mechanical stimulation (GO: 0009612) was calculated using the random walk with restart algorithm, and top 10 were selected as candidate genes for subsequent analysis. (b) In the candidate gene set, gene groups were identified by the “Correlation AnalyzeR” R package as the members of the set who share correlations in common that are not shared with other members, thereby classifying genes with common biological functions. (c) The correlation-based gene set enrichment analysis results of genes in each cluster.

1240 enrichment results of biological processes (BPs) were identified through the GO analysis.  Figure 5(a) shows the top 10 enriched BPs. Three BPs caught our attention, namely, responses to mechanical stimuli (*p* = 1.46e^−10^), reactive oxygen species metabolic process (*p* = 3.28e^−10^), and reactive oxygen species biosynthetic process (*p* = 5.65e^−10^), as mechanical stress and oxidative stress are considered to promote cellular senescence and serve as risk factors for the development of IDD [38]. We merged the reactive oxygen species metabolic process and reactive oxygen species biosynthetic process as the reactive oxygen species synthesis and metabolism process. Figures 5(b) and 5(c) show the interaction between the proteins enriched in the above two BPs. Both BPs were enriched in 11 targets, respectively. To further explore how DJD regulates the cellular response to mechanical stress, we used the target genes enriched in this BP as seed genes and the random walk algorithm with restart to calculate its diffusion score in the PPI network composed of 216 mechanical stress-related genes. Then, the top 10 genes with scores were selected as candidate genes for subsequent analysis: FOS, RETN, TNF, PTGS2, EDN1, MMP2, TLR4, JUN, NRXN1, and MAPK3 (Figure 6(a)). Notably, PPARG is the highest scoring gene, indicating its essential role in DJD regulating cellular mechanical stress responses. Cluster analysis based on the co-expression correlation of genes in cartilage tissue divides candidate genes into 4 clusters (Figure 6(b)), each of which may synergistically play a specific role in the cellular response to mechanical stress. The genes in cluster 1 include MMP2, AKT1, CASP8, and CTNNB1, and analysis found that they are all significantly associated with “laminin interaction” (Figure 6(c)). The second cluster genes include COL1A1, GJA1, TLR4, DRD2, EDN1, MAPK3, and HR2A. Although COL1A1 and HR2A are associated with extracellular matrix composition, no typical biological process is significantly associated with them. The third cluster genes are related to the regulation of ion channel activity (Figure 6(c)). COL3A1 and MPO are related to the regulation of potassium ion channel activity, while RETN and NRXN1 are related to the regulation of extracellular ligand-gated channel activity. The fourth cluster genes are significantly associated with transcriptional regulation, including PPARG, JUN, PTGS2, and FOS (Figure 6(c)). Using the same approach, we further explored how DJD regulates the processing of reactive oxygen species. The top 10 genes include NOS3, CYBB, NOX4, CAT, DUOXA1, NOX1, NCF1, CYBA, NOX5, and NCF4, while DUOX2 is the gene with the highest score (Figure 7(a)). These genes are grouped into 3 clusters (Figure 7(b)), related to the synthesis and metabolism of oxidative products in the cellular respiratory chain and mitochondria, the cyclooxygenase P450 pathway, fatty acid oxidation, Rho/Ras protein activation, and hypoxia targets of HIF1A and FOXA2 (Figure 7(c)).

### 3.6. DJD Affects Cellular Inflammatory Responses, Autophagy, and Apoptosis

In addition to mechanical stress and oxidative stress, inflammation also plays an essential role in IDD, while apoptosis and autophagy regulate the clearance process of aging cells. Therefore, we used the method proposed by Barabasi et al. to evaluate the relationship between the target genes of DJD and the above gene groups in the context of the human PPI network and found that DJD may involve in regulating cellular inflammatory responses (*s*_AB_ = −0.19), autophagy (*s*_AB_ = −0.20), and apoptosis (*s*_AB_ = −0.19) (Table 3).

### 3.7. KEGG Pathway Analysis

**Figure 8:**
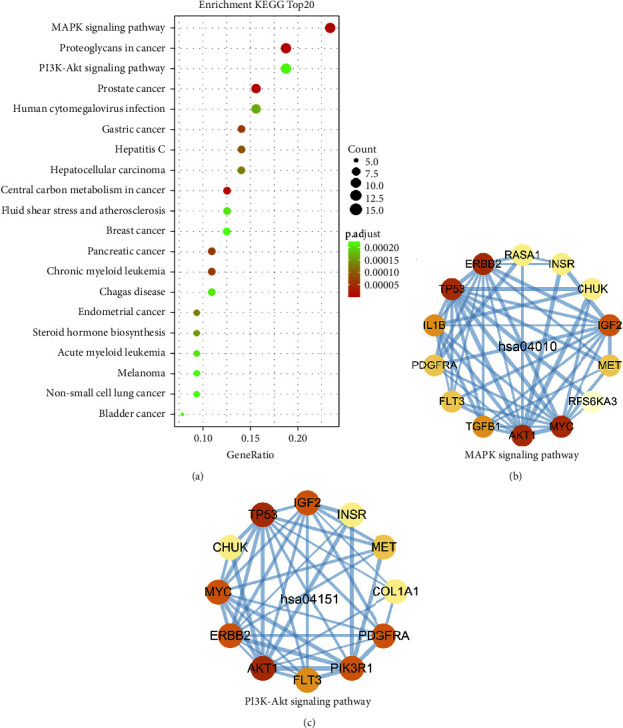
KEGG enrichment analysis results of common target genes. (a) The top 20 KEGG enrichment analysis results. (b) The PPI network of common target genes enriched in the MAPK signaling pathway. (c) The PPI network of common target genes enriched in the PI3K/AKT signaling pathway.

The KEGG pathway analysis was conducted, and a pathway with a *p* value <0.005 was considered significantly enriched. Finally, 76 significantly enriched pathways were screened out.  Figure 8(a) shows the top 20 pathways, and more information is detailed in Table S6. The most significantly enriched pathway is the MAPK pathway (Figure 8(b), Figure S2), and the PI3K/AKT pathway is also significantly enriched (Figure 8(c), Figure S3). Interestingly, many other significantly enriched pathways intercommunicate with PI3K/AKT pathway (Table S6), including MAPK pathway, cell cycle, apoptosis, FoxO pathway, and toll-like receptor pathway [39]. The above suggests that the MAPK and PI3K/AKT pathways may play a vital role in treating IDD by DJD. In addition, it is worth noting that CHUK (IKK-A), one of the targets of DJD, acts as part of the canonical IKK complex which is a hub for PI3K/AKT pathway to connect with the NF-*κ*B pathway [40].

## 4. Discussion

IDD was believed to be DDD's pathological basis [2]. A series of factors, such as mechanical stress, oxidative stress, and inflammation, promote the senescence of NPCs [38]. DJD is a traditional Chinese medicine formula with a history of thousands of years, and it has been used to treat osteoarthritis in the past due to its anti-autophagy and anti-inflammatory effects [10]. Recent studies have also found the potential of DJD to treat IDD, such as activating autophagy and significantly reducing apoptosis and matrix degeneration in nucleus pulposus cells and inhibiting inflammation [11–14]. There are many botanical drugs in DJD, forming a compound library that synergistically exerts biological functions. Among the compounds of DJD, Quercetin and Kaempferol are assigned a central position in the treatment of IDD because they form the densest associations with IDD-related targets and target crucial targets (such as AKT1, PIK3R1, TP53, CHUK, IGF-1, ERBB2, MYC, IL1B, and CAV1). AKT1 was reported to involve in the regulation of autophagy of degenerated NPCs and extracellular matrix metabolism. Moreover, cellular senescence is the primary pathological process of IDD, and Akt can phosphorylate and inhibit p27 and p21, which are closely related to cellular senescence. ERBB2 is involved in regulating the extracellular matrix metabolism of disc, while IL1BR, IGF-1, and CTNNB1 are mainly related to the regulation of the inflammatory response in degenerative discs [41–45]. TP53 is closely related to cell senescence. p53-p21 pathway and p16-Rb pathway are the most important signaling pathways that mediate most cellular senescence phenomena [46]. The above results indicate that these key targets play an important role in DJD treatment of IDD.

Based on the predicted toxicity parameters, DJD has a relatively low risk in terms of hepatotoxicity, which was confirmed in a previous study. A clinical trial that evaluated the possible liver and kidney damage of DJD revealed that no significant changes in liver or kidney functions and the severe incidence of adverse events were observed during the 4 weeks of administration of DJD [47]. However, the risks of DJD in terms of cytotoxicity, immunotoxicity, and cardiotoxicity require more consideration. While there is currently insufficient evidence to suggest that DJD may pose a safety risk, understanding the effects of the various botanical drugs in DJD can help reduce the risk and provide further insight into its treatment mechanism.

A previous study has identified the MAPK pathway as a possible mechanism for DJD to treat IDD [14]. Interestingly, this study also revealed that the MAPK pathway has a prominent performance in DJD treatment of IDD (Figure 8). Furthermore, this study reveals the mechanism by which DJD can treat IDD by regulating the MAPK pathway: Quercetin targets AKT1, TP53, ERBB2, MYC, IL1B, CHUK, IGF2, RASA1, MET, and RPS6KA3; Kaempferol targets AKT1, INSR, and FLT3; Beta-Sitosterol targets TGFB1; Yangambin targets CACNA1S; and Methylicosa-11,19-dienoate targets PDGFRA. The above targets are mainly involved in the classical MAP kinase signaling pathway (IGF2, CACNA1S, INSR, FLT3, ERBB2, MET, PDGFRA, RASA1, CHUK, RPS6KA3, and MYC) represented by Ras and the JNK and p38 kinase pathway (IL1B, TGFB1, AKT1, and TP53) (Figure S2). The physiological effects of the two MAP kinase pathways are mainly related to inflammation and apoptosis [48, 49]. In addition, the analysis of network proximity and transcription factor regulatory network also indicates that the targets of DJD have roles in regulating inflammation and autophagy. This suggests that the MAPK signaling pathway may be an important approach for DJD to achieve the regulation of inflammation and apoptosis to treat IDD. Quercetin and Kaempferol are key compounds that DJD depends on to regulate the MAPK pathway because they have more targets on the pathway and their targets cover some important members, such as AKT1, TP53, MYC, and CHUK. The above results further confirmed and supplemented the specific molecular mechanism by which DJD regulates the MAPK pathway in the treatment of IDD. The NF-*κ*B pathway has also been shown to play a role in the DJD treatment of IDD in a previous study [13]. CHUK (IKK-A) acts as part of the canonical IKK complex in the conventional pathway of NF-kappa-B activation and phosphorylates inhibitors of NF-kappa-B on serine residues [40]. Moreover, CHUK is the target of Quercetin, which provides a potential mechanism by which DJD regulates the NF-*κ*B pathway. In addition, AKT1 is involved in the phosphorylation of CHUK and has an important role in NF-*κ*B-dependent regulation of gene transcription (Figure S3) [40]. Therefore, AKT1/CHUK is also a possible pathway for DJD to regulate NF-*κ*B pathway. NF-*κ*B pathway is involved in the pathological process of a variety of inflammatory diseases. Activation of NF-*κ*B pathway targets downstream inflammatory cytokines and promotes intervertebral disc degeneration [50]. Therefore, the NF-*κ*B pathway may play a role in the regulation of inflammation in the treatment of IDD by DJD. The PI3K/AKT pathway has attracted our great attention. Both core members of this pathway are targets of DJD (Kaempferol, Beta-Carotene, Quercetin, Baicalein, and Wogonin target AKT1; Quercetin targets PIK3R1). Possible biological effects induced by the PI3K/AKT signaling pathway in IDD include increasing extracellular matrix content, anti-apoptosis, induction or inhibition of autophagy to prevent IDD, and anti-oxidative stress [51]. Besides, more importantly, PI3K/AKT pathway has broad associations with other pathways that were significantly enriched for common target genes [39]. Interestingly, many related pathways (cell cycle, apoptosis, and FoxO signaling pathway) play important roles in cellular senescence, which was considered the pathological basis of IDD, by regulating cell cycle, apoptosis, autophagy, and oxidative stress 6, 55. The above results suggest that DJD may target genes located in a network consisting of the PI3K/AKT pathway and its related pathways, especially the MAPK pathway, which contains the most common target genes, to regulate a series of biological processes (such as cellular senescence, inflammatory response, and oxidative stress) to affect the aging process of the intervertebral disc [48, 49, 51, 52].

Functional classification analysis of common target genes revealed that DJD might be involved in regulating oxidative stress and kinases and transcription factors (Figure 2(a)). The regulatory role of DJD in the cellular oxidative stress response is also verified by GO analysis (Figure 5). Furthermore, GO analysis also suggests that DJD can modulate cellular responses to mechanical stress (Figure 5). Moreover, further analysis shows that DJD may act on extracellular matrix components, especially laminin (Figure 6(c)), which not only plays an important role in mechanical stress signal transduction but also regulates the synthesis and metabolism of extracellular matrix [43]. In addition, MMP1 and MMP11, the targets of DJD, are key enzymes in extracellular matrix degradation, affecting the balance of extracellular matrix synthesis and catabolism in nucleus pulposus [36]. Imbalances in extracellular matrix synthesis and metabolism in IDD lead to its reduction, which further develops with severe consequences, including rupture of the annulus fibrosus and destruction of NPCs [37]. Therefore, the possible regulatory role of DJD on the extracellular matrix is of great significance for delaying IDD. In addition, regulating ion channels is a key process in cellular mechanical stress signal transduction [53], and DJD may be involved in regulating ion channels, especially potassium channels. Moreover, DJD may also play a role in the cellular response to mechanical stress by regulating transcription (Figure 6(c)). As for the regulation of oxidative stress by DJD, this study shows that DJD may play a role in the synthesis and metabolism of reactive oxygen species in the respiratory chain and mitochondria and fatty acid oxidation and may also be involved in the cyclooxygenase P450 pathway and regulation of Rho and Ras protein activation. The cytochrome P450 cyclooxygenase pathway mediates the metabolism of Arachidonic acid and is involved in BPs such as oxidative stress, inflammation, immunity, apoptosis, and proliferation [54]. Rho protein and Ras protein are GTPases. Rho kinase (ROCK) is an effector of Rho. Its upregulation induces oxidative stress [55]. Besides the regulatory role of DJD in cellular responses to mechanical and oxidative stress, the network proximity index analysis also indicates that DJD plays a role in cellular inflammatory responses, apoptosis, and autophagy (Table 3). Furthermore, HIF1A, PARP1, RELA, and AR function as transcriptional regulators and targets of DJD and play roles in apoptosis, inflammatory responses, hypoxia, cellular senescence, and mechanical stress stimulation (Table 1). Overall, our findings above support the role of DJD in several pathological processes in IDD, including oxidative stress, mechanical stress, inflammation, extracellular matrix synthesis and metabolism, apoptosis, and autophagy.

## 5. Conclusion

AKT1, PIK3R1, TP53, MYC, CTNNB1, ALB, NR3C1, IL1B, ERBB2, CAV1, AR, IGF2, and ESR1 are crucial targets of DJD in the treatment of IDD. DJD is involved in multiple physiological and pathological processes of IDD, mainly including the regulation of mechanical stress, oxidative stress, inflammation, and autophagy. MAPK pathway, PI3K/AKT pathway, and NF-*κ*B pathway play a pivotal role in DJD treatment of IDD. Quercetin and Kaempferol are the key compounds of DJD in the treatment of IDD.

## Figures and Tables

**Table 1:** The biological processes associated with IDD that are regulated by HIF1A, RARP, RELA, and AR.

Transcription factor	Biological process	*p* value^*∗*^
HIF1A	Response to hypoxia	5.30209*E* − 21
	Anti-apoptosis	4.15541*E* − 07
	Aging	5.60851*E* − 05
	Positive regulation of inflammatory response	0.000132004

RARP	Anti-apoptosis	6.35527*E* − 05
	Regulation of cell cycle	0.000381163
	Cellular senescence	0.000418457
	Negative regulation of inflammatory response	0.002881088
	Response to mechanical stimulus	0.003308069

RELA	Inflammatory response	3.54528*E* − 37
	Response to hypoxia	1.00592*E* − 27
	Anti-apoptosis	1.53185*E* − 21
	Cellular response to mechanical stimulus	3.79238*E* − 12
	Aging	4.88522*E* − 08

AR	Anti-apoptosis	1.93119*E* − 08
	Response to mechanical stimulus	3.11925*E* − 05
	Regulation of cell cycle	0.000109675

^*∗*^ *p* values are calculated with the hypergeometric test.

**Table 2:** Results of molecular docking.

Gene	Molecular ligand	Binding free energy (kcal/mol)	Inhibition constant (*μ*mol/ml)
AKT1	Kaempferol	−7.04	6.88
AKT1	Quercetin	−7.23	4.97
PIK3R1	Quercetin	−6.12	32.84
ESR1	Myricanone	−8.16	1.04
MYC	Quercetin	−5.96	42.99
NR3C1	Wallichilide	−10.44	0.02
IL1B	Quercetin	−8.28	0.84
TP53	Quercetin	−6.67	12.95
ERRB2	Quercetin	−5.12	175.26
CAV1	Quercetin	−5.5	92.39
AR	Kaempferol	−8.26	0.88
IGF2	Quercetin	−5.15	167.49
ALB	Beta-Carotene	−12.61	0.57 × 10^−3^
CTNNB1	Beta-Carotene	−8.43	0.66

**Table 3:** The network proximity index values between the target genes of DJD and the corresponding gene groups.

Gene group	*s*_AB_
Autophagy	−0.20
Apoptosis	−0.19
Inflammatory response	−0.19
Random control	0.05

## Data Availability

The data generated or analyzed in this study are included in the article and its supplementary material; further inquiries can be directed to the corresponding author.

## Supplementary Materials

Supplementary Materials Table S1 records Lipinski's rule of five (RO5). Table S2 details the renaming results of the compounds in DJD. Table S3 shows the information on receptor proteins and docking sites. Table S4 lists the 67 DJD compounds that we screened for oral bioavailability and therapeutic potential. Table S5 details the protein-ligand interaction information. Table S6 exhibits the results of KEGG pathway analysis of common targets. Figure S1 shows the protein complex or functional module in the PPI network of common targets. Figure S2 shows the target genes of DJD involved in MAPK signaling pathway. Figure S3 shows the target genes of DJD involved in PI3K/AKT signaling pathway.
