# Supplementary material for: A Study Based on Network Pharmacology Decoding the Multi-Target Mechanism of Duhuo Jisheng Decoction for the Treatment of Intervertebral Disc Degeneration
Source: Comput Intell Neurosci. 2023 May 28;2023:7091407. doi: 10.1155/2023/7091407 (PMC10243954; doi:10.1155/2023/7091407)
Supplement: Supplementary Materials — Table S1 records Lipinski's rule of five (RO5). Table S2 details the renaming results of the compounds in DJD. Table S3 shows the information on receptor proteins and docking sites. Table S4 lists the 67 DJD compounds that we screened for oral bioavailability and therapeutic potential. Table S5 details the protein-ligand interaction information. Table S6 exhibits the results of KEGG pathway analysis of common targets. Figure S1 shows the protein complex or functional module in the PPI network of common targets. Figure S2 shows the target genes of DJD involved in MAPK signaling pathway. Figure S3 shows the target genes of DJD involved in PI3K/AKT signaling pathway. [file 7091407.f1.zip › Supplementary Material (2).docx]

**Table S1.** Lipinski’s rule of five (RO5)

| **Structural parameters** | **Cutoff value** |
| --- | --- |
| Molecule weight (MW) | < 500 |
| Number of hydrogen bond donors (Hdon) | < 5 |
| Number of hydrogen bond  acceptors (Hacc) | ≤ 10 |
| Lipid-water partition coefficient (LogP) | ≤ 5 |
| Number of rotatable bonds (Rbon) | ≤ 10 |

**Table S2.** Rename results of the compounds in DJD

| **Compounds** | **Renamed name** | **Botanical drugs** |
| --- | --- | --- |
| paeoniflorgenone | BS1 | Paeoniae Radix Alba |
| (3S,5R,8R,9R,10S,14S)-3,17-dihydroxy-4,4,8,10,14-pentamethyl-2,3,5,6,7,9-hexahydro-1H-cyclopenta[a]phenanthrene-15,16-dione | BS2 | Paeoniae Radix Alba |
| mairin | A | Paeoniae Radix Alba，Eucommiae Cortex |
| beta-sitosterol | B | Paeoniae Radix Alba,Angelicae Sinensis Radix,Radix Angelicae Biseratae,Eucommiae Cortex,Saposhnikoviae Radix,Achyranthis Bidentatae Radix,Gentiana Macrophylla Pall,Panax Ginseng C. A. Mey. |
| sitosterol | C | Paeoniae Radix Alba,Chuanxiong Rhizoma,Saposhnikoviae Radix,Gentiana Macrophylla Pall,Herba Taxilli,Rehmanniae Radix Praeparata |
| kaempferol | D | Paeoniae Radix Alba,Eucommiae Cortex,Achyranthis Bidentatae Radix,Panax Ginseng C. A. Mey.,Asari Radix Et Rhizoma |
| mandenol | E | Chuanxiong Rhizoma,Saposhnikoviae Radix |
| myricanone | CX1 | Chuanxiong Rhizoma |
| perlolyrine | CX2 | Chuanxiong Rhizoma |
| wallichilide | CX3 | Chuanxiong Rhizoma |
| stigmasterol | F | Angelicae Sinensis Radix,Achyranthis Bidentatae Radix,Panax Ginseng C. A. Mey.,Rehmanniae Radix Praeparata |
| angelicone | DH1 | Radix Angelicae Biseratae |
| O-Acetylcolumbianetin | DH2 | Radix Angelicae Biseratae |
| erythraline | DZ1 | Eucommiae Cortex |
| 3-beta-Hydroxymethyllenetanshiquinone | DZ2 | Eucommiae Cortex |
| yangambin | DZ3 | Eucommiae Cortex |
| (-)-Tabernemontanine | DZ4 | Eucommiae Cortex |
| cyclopamine | DZ5 | Eucommiae Cortex |
| Dehydrodiconiferyl alcohol 4,gamma'-di-O-beta-D-glucopyanoside_qt | DZ6 | Eucommiae Cortex |
| Cinchonan-9-al, 6'-methoxy-, (9R)- | DZ7 | Eucommiae Cortex |
| helenalin | DZ8 | Eucommiae Cortex |
| 4-[(2S,3R)-5-[(E)-3-hydroxyprop-1-enyl]-7-methoxy-3-methylol-2,3-dihydrobenzofuran-2-yl]-2-methoxy-phenol | DZ9 | Eucommiae Cortex |
| quercetin | G | Eucommiae Cortex,Achyranthis Bidentatae Radix,Herba Taxilli |
| liriodendrin_qt | DZ10 | Eucommiae Cortex |
| beta-carotene | DZ11 | Eucommiae Cortex |
| (E)-3-[4-[(1R,2R)-2-hydroxy-2-(4-hydroxy-3-methoxy-phenyl)-1-methylol-ethoxy]-3-methoxy-phenyl]acrolein | DZ12 | Eucommiae Cortex |
| syringetin | DZ13 | Eucommiae Cortex |
| wogonin | H | Saposhnikoviae Radix,Achyranthis Bidentatae Radix |
| 5-O-Methylvisamminol | FF1 | Saposhnikoviae Radix |
| ledebouriellol | FF2 | Saposhnikoviae Radix |
| divaricatol | FF3 | Saposhnikoviae Radix |
| methyl icosa-11,14-dienoate | FF4 | Saposhnikoviae Radix |
| (2R,3R)-3-(4-hydroxy-3-methoxy-phenyl)-5-methoxy-2-methylol-2,3-dihydropyrano[5,6-h][1,4]benzodioxin-9-one | FF5 | Saposhnikoviae Radix |
| (2R)-2-[(3S,5R,10S,13R,14R,16R,17R)-3,16-dihydroxy-4,4,10,13,14-pentamethyl-2,3,5,6,12,15,16,17-octahydro-1H-cyclopenta[a]phenanthren-17-yl]-6-methylhept-5-enoic acid | FL1 | Saposhnikoviae Radix |
| trametenolic acid | FL2 | Saposhnikoviae Radix |
| cerevisterol | FL3 | Saposhnikoviae Radix |
| ergosta-7,22E-dien-3beta-ol | FL4 | Saposhnikoviae Radix |
| Ergosterol peroxide | FL5 | Saposhnikoviae Radix |
| hederagenin | FL6 | Saposhnikoviae Radix |
| poriferasta-7,22E-dien-3beta-ol | NX1 | Achyranthis Bidentatae Radix |
| berberine | NX2 | Achyranthis Bidentatae Radix |
| coptisine | NX3 | Achyranthis Bidentatae Radix |
| delta 7-stigmastenol | NX4 | Achyranthis Bidentatae Radix |
| baicalein | NX5 | Achyranthis Bidentatae Radix |
| epiberberine | NX6 | Achyranthis Bidentatae Radix |
| Inophyllum E | NX7 | Achyranthis Bidentatae Radix |
| spinasterol | NX8 | Achyranthis Bidentatae Radix |
| beta-daucosterol_qt | NX9 | Achyranthis Bidentatae Radix |
| diop | RS1 | Panax Ginseng C. A. Mey. |
| inermin | RS2 | Panax Ginseng C. A. Mey. |
| aposiopolamine | RS3 | Panax Ginseng C. A. Mey. |
| deoxyharringtonine | RS4 | Panax Ginseng C. A. Mey. |
| dianthramine | RS5 | Panax Ginseng C. A. Mey. |
| arachidonate | RS6 | Panax Ginseng C. A. Mey. |
| frutinone A | RS7 | Panax Ginseng C. A. Mey. |
| ginsenoside-Rh4_qt | RS8 | Panax Ginseng C. A. Mey. |
| girinimbin | RS9 | Panax Ginseng C. A. Mey. |
| panaxadiol | RS10 | Panax Ginseng C. A. Mey. |
| suchilactone | RS11 | Panax Ginseng C. A. Mey. |
| alexandrin_qt | RS12 | Panax Ginseng C. A. Mey. |
| fumarine | RS13 | Panax Ginseng C. A. Mey. |
| 4,9-dimethoxy-1-vinyl-$b-carboline | XX1 | Asari Radix Et Rhizoma |
| caribine | XX2 | Asari Radix Et Rhizoma |
| cryptopin | XX3 | Asari Radix Et Rhizoma |
| sesamin | XX4 | Asari Radix Et Rhizoma |
| [(1S)-3-[(E)-but-2-enyl]-2-methyl-4-oxo-1-cyclopent-2-enyl] (1R,3R)-3-[(E)-3-methoxy-2-methyl-3-oxoprop-1-enyl]-2,2-dimethylcyclopropane-1-carboxylate | XX5 | Asari Radix Et Rhizoma |
| (3S)-7-hydroxy-3-(2,3,4-trimethoxyphenyl)chroman-4-one | XX6 | Asari Radix Et Rhizoma |
| ZINC05223929 | XX7 | Asari Radix Et Rhizoma |

**Table S3.** Information on receptor proteins and docking sites

| **Gene** | **PDB ID** | **Method and resolution** | **Mocular ligand** | **Grid Box (X, Y, Z)** |
| --- | --- | --- | --- | --- |
| AKT1 | 4GV1 | X-RAY, 0.98 Å | Kaempferol | Size: 32,44,52  Center coordinate:  -20.302, 5.763, 11.822 |
| AKT1 | 4GV1 | X-RAY, 0.98 Å | Quercetin | Size: 32,44,52  Center coordinate:  -20.302, 5.763, 11.822 |
| PIK3R1 | 2IUH | X-RAY, 2.00 Å | Quercetin | Size: 48,48,54  Center coordinate:  21.32, 11.245, 15.199) |
| ESR1 | 2OUZ | X-RAY, 2.00 Å | Myricanone | Size: 64, 66, 60  Center coordinate:  28.33, 4.551, 3.222 |
| MYC | 5I50 | X-RAY, 2.68 Å | Quercetin | Size: 48, 52, 60  Center coordinate:  76.325, 67.629, 39.591 |
| NR3C1 | 4UDD | X-RAY, 1.80 Å | Wallichilide | Size: 82, 104, 68  Center coordinate:  38.769, 12.426, 10.792 |
| IL1B | 5R85 | X-RAY, 1.44 Å | Quercetin | Size: 54, 42, 34  Center coordinate:  38.769, 6.505, 71.073 |
| TP53 | 3KMD | X-RAY, 2.15 Å | Quercetin | Size: 62, 50, 54  Center coordinate:  -2.361, 21.001, -25.472 |
| ERBB2 | 4HRN | X-RAY, 2.65 Å | Quercetin | Size: 64, 64, 72  Center coordinate:  23.149, 5.841, -21.572 |
| CAV1 | 7SC0 | ELECTRON MICROSCOPY, 3.4 Å | Quercetin | Size: 66, 86, 52  Center coordinate:  145.707, 5.841, -21.572 |
| AR | 2PIW | X-RAY, 2.58 Å | Kaempferol | Size: 64, 80, 68  Center coordinate:  27.072, 4.657, 4.915 |
| IGF2 | 3KR3 | X-RAY, 2.2 Å | Quercetin | Size: 44, 32, 36  Center coordinate:  11.222, 27.514,6.697 |
| ALB | 6YG9 | X-RAY, 1.89 Å | Beta-carotene | Size: 112, 92, 94  Center coordinate:  57.357, -2.806, 26.019 |
| CTNNB1 | 7AFW | X-RAY,1.81 Å | Beta-carotene | Size: 42, 68, 52  Center coordinate:  75.13, -34.912, 35.769 |

**Table S4.** The 67 screened compounds of DJD with oral bioavailability and therapeutic potential

| Chinese name of Herb (abbreviation) | Latin Name of Herb ^#^ | Compounds |
| --- | --- | --- |
| Duhuo (DH) | Angelica biserrata (R.H.Shan & C.Q.Yuan) C.Q.Yuan & R.H.Shan | Angelicone, O-Acetylcolumbianetin, beta-sitosterol |
| Sangjisheng (SJ) | Taxillus sutchuenensis (Lecomte) Danser | Quercetin, sitosterol |
| Qinjiao (QJ) | Gentiana Macrophylla Pall | beta-sitosterol, sitosterol |
| Fangfeng (FF) | Saposhnikovia divaricata (Turcz.) Schischk | Wogonin, 5-O-Methylvisamminol, ledebouriellol, divaricatol,Mandenol, sitosterol, beta-sitosterol, methyl icosa-11,14-dienoate, 1* |
| Xixing (XX) | Asarum sieboldii Miq | 4,9-dimethoxy-1-vinyl-$b-carboline, Caribine, Cryptopin, sesamin, 2*, 3*, kaempferol, ZINC05223929 |
| Danggui (DG) | Angelica sinensis (Oliv.) Diels | Stigmasterol, beta-sitosterol |
| Chuanxiong (CX) | Ligusticum chuanxiong S.H.Qiu, Y.Q.Zeng, K.Y.Pan, Y.C.Tang & J.M.Xu | Mandenol, Myricanone, Perlolyrine, wallichilide, sitosterol |
| Shudihuang (SD) | Rehmannia glutinosa (Gaertn.) DC | Sitosterol, Stigmasterol |
| Baishao (BS) | Paeonia laciniata Pall | paeoniflorin_qt, Mairin, beta-sitosterol, sitosterol, kaempferol |
| Rougui (RG) | Cinnamomum cassia (L.) J.Presl | - |
| Fuling (FL) | Poria Cocos (Schw.) Wolf. | 4*, trametenolic acid, 7,9(11)-dehydropachymic acid, Cerevisterol, 5*, ergosta-7,22E-dien-3beta-ol, Ergosterol peroxide, 6*, 3beta-Hydroxy-24-methylene-8-lanostene-21-oic acid, pachymic acid, Poricoic acid A, Poricoic acid B, poricoic acid C, hederagenin, dehydroeburicoic acid |
| Duzhong (DZ) | Eucommia ulmoides Oliv | 7*, liriodendrin_qt, quercetin, beta-carotene, 8*, Syringetin |
| Niuxi (NX) | Achyranthes bidentata Blume | berberine, coptisine, wogonin, delta 7-stigmastenol, baicalein, epiberberine, beta-sitosterol, Inophyllum E, kaempferol, Spinasterol, Stigmasterol, beta-daucosterol_qt, quercetin |
| Rensheng (RS) | Panax Ginseng C. A. Mey. | Diop, Stigmasterol, beta-sitosterol, Inermin, kaempferol, Chrysanthemaxanthin, Aposiopolamine, Celabenzine, Deoxyharringtonine, Dianthramine, arachidonate, Frutinone A, Ginsenoside-Rh4_qt, Girinimbin, Gomisin B, malkangunin, Panaxadiol, suchilactone, alexandrin_qt, ginsenoside Rg5_qt, Fumarine |

^#^ Complete Latin plant names are provided according to the criteria on the website (www.theplantlist.org)

1*: (2R,3R)-3-(4-hydroxy-3-methoxy-phenyl)-5-methoxy-2-methylol-2,3-dihydropyrano[5,6-h][1,4]benzodioxin-9-one; 2*: [(1S)-3-[(E)-but-2-enyl]-2-methyl-4-oxo-1-cyclopent-2-enyl](1R,3R)-3-[(E)-3-methoxy-2-methyl-3-oxoprop-1-enyl]-2,2-dimethylcyclopropane-1-carboxylate; 3*: (3S)-7-hydroxy-3-(2,3,4-trimethoxyphenyl)chroman-4-one; 4*: (2R)-2-[(3S,5R,10S,13R,14R,16R,17R)-3,16-dihydroxy-4,4,10,13,14-pentamethyl-2,3,5,6,12,15,16,17-octahydro-1H-cyclopenta[a]phenanthren-17-yl]-6-methylhept-5-enoicacid; 5*: (2R)-2-[(3S,5R,10S,13R,14R,16R,17R)-3,16-dihydroxy-4,4,10,13,14-pentamethyl-2,3,5,6,12,15,16,17-octahydro-1H-cyclopenta[a]phenanthren-17-yl]-5-isopropyl-hex-5-enoicacid; 6*: (2R)-2-[(5R,10S,13R,14R,16R,17R)-16-hydroxy-3-keto-4,4,10,13,14-pentamethyl-1,2,5,6,12,15,16,17-octahydrocyclopenta[a]phenanthren-17-yl]-5-isopropyl-hex-5-enoicacid; 7*: 4-[(2S,3R)-5-[(E)-3-hydroxyprop-1-enyl]-7-methoxy-3-methylol-2,3-dihydrobenzofuran-2-yl]-2-methoxy-phenol; 8* : (E)-3-[4-[(1R,2R)-2-hydroxy-2-(4-hydroxy-3-methoxy-phenyl)-1-methylol-ethoxy]-3-methoxy-phenyl]acrolein

**Table S5.** Protein-ligand inteaction infomation

| **Protein-Ligand** | **Index** | **Residue** | **AA** | **Distance** | **Type** |
| --- | --- | --- | --- | --- | --- |
| AKT1_Kaempferol | 1 | 211A | THR | 3.08 | Hydrophobic Interactions |
|  | 2 | 227A | MET | 3.84 | Hydrophobic Interactions |
|  | 3 | 438A | PHE | 3.04 | Hydrophobic Interactions |
|  | 1 | 156A | LEU | 1.94 (H-A), 2.76 (D-A) | Hydrogen Bonds |
|  | 2 | 230A | ALA | 2.31 (H-A), 3.31 (D-A) | Hydrogen Bonds |
|  | 3 | 292A | ASP | 2.08 (H-A), 3.08 (D-A) | Hydrogen Bonds |
|  | 4 | 292A | ASP | 2.11 (H-A), 3.07 (D-A) | Hydrogen Bonds |
| AKT1_Quercetin | 1 | 164A | VAL | 3.93 | Hydrophobic Interactions |
|  | 2 | 177A | ALA | 3.55 | Hydrophobic Interactions |
|  | 3 | 234A | GLU | 3.01 | Hydrophobic Interactions |
|  | 4 | 438A | PHE | 2.97 | Hydrophobic Interactions |
|  | 5 | 438A | PHE | 3.83 | Hydrophobic Interactions |
|  | 6 | 442A | PHE | 3.49 | Hydrophobic Interactions |
|  | 1 | 228A | GLU | 2.61 (H-A), 3.29 (D-A) | Hydrogen Bonds |
|  | 2 | 230A | ALA | 2.2 (H-A), 3.18 (D-A) | Hydrogen Bonds |
|  | 3 | 234A | GLU | 2.06 (H-A), 3.07 (D-A) | Hydrogen Bonds |
|  | 4 | 234A | GLU | 2.08 (H-A), 3.05 (D-A) | Hydrogen Bonds |
|  | 5 | 292A | ASP | 3.16 (H-A), 3.74 (D-A) | Hydrogen Bonds |
|  | 6 | 437A | TYR | 2.11 (H-A), 2.93 (D-A) | Hydrogen Bonds |
|  | 7 | 439A | ASP | 1.83 (H-A), 2.71 (D-A) | Hydrogen Bonds |
| PIK3R1_Quercetin | 1 | 58A | LYS | 3.66 | Hydrophobic Interactions |
|  | 2 | 58A | LYS | 3.14 | Hydrophobic Interactions |
|  | 3 | 71A | PHE | 3.06 | Hydrophobic Interactions |
|  | 4 | 95A | TYR | 3.96 | Hydrophobic Interactions |
|  | 1 | 58A | LYS | 2.75 (H-A), 3.57 (D-A) | Hydrogen Bonds |
|  | 2 | 59A | LEU | 1.94 (H-A), 2.38 (D-A) | Hydrogen Bonds |
|  | 3 | 61A | LYS | 2.19 (H-A), 3.14 (D-A) | Hydrogen Bonds |
|  | 4 | 61A | LYS | 2.54 (H-A), 3.42 (D-A) | Hydrogen Bonds |
|  | 5 | 96A | ASN | 2.56 (H-A), 2.99 (D-A) | Hydrogen Bonds |
|  | 6 | 96A | ASN | 2.00 (H-A), 2.96 (D-A) | Hydrogen Bonds |
|  | 7 | 98A | LYS | 3.15 (H-A), 3.66 (D-A) | Hydrogen Bonds |
| ESR1_Myricanone | 1 | 383A | TRP | 3.12 | Hydrophobic Interactions |
|  | 2 | 525A | LEU | 3.47 | Hydrophobic Interactions |
|  | 3 | 525A | LEU | 3.48 | Hydrophobic Interactions |
|  | 4 | 533A | VAL | 3.78 | Hydrophobic Interactions |
|  | 5 | 536A | LEU | 3.91 | Hydrophobic Interactions |
|  | 6 | 536A | LEU | 3.35 | Hydrophobic Interactions |
|  | 7 | 536A | LEU | 3.63 | Hydrophobic Interactions |
|  | 8 | 539A | LEU | 3.33 | Hydrophobic Interactions |
|  | 1 | 526A | TYR | 2.99 (H-A), 3.94 (D-A) | Hydrogen Bonds |
|  | 2 | 529A | LYS | 1.68 (H-A), 2.58 (D-A) | Hydrogen Bonds |
|  | 3 | 534A | VAL | 2.06 (H-A), 2.97 (D-A) | Hydrogen Bonds |
|  | 4 | 536A | LEU | 1.9 (H-A), 2.74 (D-A) | Hydrogen Bonds |
| MYC_Quercetin | 1 | 932A | GLU | 3.31 | Hydrophobic Interactions |
|  | 1 | 925A | ARG | 1.82 (H-A), 2.49 (D-A) | Hydrogen Bonds |
|  | 2 | 925A | ARG | 2.74 (H-A), 3.29 (D-A) | Hydrogen Bonds |
|  | 3 | 926A | ASP | 2.11 (H-A), 2.94 (D-A) | Hydrogen Bonds |
|  | 4 | 928A | ILE | 2.77 (H-A), 3.33 (D-A) | Hydrogen Bonds |
|  | 5 | 932A | GLU | 1.83 (H-A), 2.79 (D-A) | Hydrogen Bonds |
| NR3C1_Wallichilide | 1 | 604A | MET | 3.44 | Hydrophobic Interactions |
|  | 2 | 608A | LEU | 3.47 | Hydrophobic Interactions |
|  | 3 | 608A | LEU | 3.45 | Hydrophobic Interactions |
|  | 4 | 623A | PHE | 3.57 | Hydrophobic Interactions |
|  | 5 | 623A | PHE | 3.81 | Hydrophobic Interactions |
|  | 6 | 642A | GLN | 3.69 | Hydrophobic Interactions |
|  | 7 | 732A | LEU | 3.62 | Hydrophobic Interactions |
|  | 8 | 732A | LEU | 3.67 | Hydrophobic Interactions |
|  | 9 | 732A | LEU | 3.41 | Hydrophobic Interactions |
|  | 10 | 735A | TYR | 3.51 | Hydrophobic Interactions |
|  | 11 | 735A | TYR | 3.5 | Hydrophobic Interactions |
|  | 12 | 735A | TYR | 3.7 | Hydrophobic Interactions |
|  | 13 | 735A | TYR | 3.57 | Hydrophobic Interactions |
|  | 14 | 735A | TYR | 3.97 | Hydrophobic Interactions |
| IL1B_Quercetin | 1 | 24A | TYR | 3.19 | Hydrophobic Interactions |
|  | 2 | 80A | LEU | 3.94 | Hydrophobic Interactions |
|  | 1 | 25A | GLU | 3.4 (H-A), 4.09 (D-A) | Hydrogen Bonds |
|  | 2 | 26A | LEU | 1.92 (H-A), 2.85 (D-A) | Hydrogen Bonds |
|  | 3 | 26A | LEU | 2.11 (H-A), 3.02 (D-A) | Hydrogen Bonds |
|  | 4 | 26A | LEU | 1.94 (H-A), 2.85 (D-A) | Hydrogen Bonds |
|  | 5 | 74A | LYS | 2.02 (H-A), 2.77 (D-A) | Hydrogen Bonds |
|  | 6 | 82A | LEU | 2.34 (H-A), 3.11 (D-A) | Hydrogen Bonds |
|  | 7 | 132A | VAL | 3.18 (H-A), 4.01 (D-A) | Hydrogen Bonds |
| TP53_Quercetin | 1 | 113A | PHE | 3.29 | Hydrophobic Interactions |
|  | 2 | 113A | PHE | 3.3 | Hydrophobic Interactions |
|  | 1 | 113A | PHE | 1.78 (H-A), 2.79 (D-A) | Hydrogen Bonds |
|  | 2 | 113A | PHE | 2.02 (H-A), 2.81 (D-A) | Hydrogen Bonds |
|  | 3 | 268A | ASN | 2.06 (H-A), 3.05 (D-A) | Hydrogen Bonds |
|  | 4 | 269A | SER | 2.18 (H-A), 3.19 (D-A) | Hydrogen Bonds |
|  | 5 | 269A | SER | 2.13 (H-A), 4.19 (D-A) | Hydrogen Bonds |
|  | 6 | 269A | SER | 2 (H-A), 5.19 (D-A) | Hydrogen Bonds |
|  | 1 | 126A | TYR | 4.68 | π-Stacking |
|  | 2 | 126A | TYR | 4.92 | π-Stacking |
| ERBB2_Quercetin | 1 | 40A | VAL | 3.09 | Hydrophobic Interactions |
|  | 2 | 69A | ASN | 3.62 | Hydrophobic Interactions |
|  | 1 | 34A | MET | 2.07 (H-A), 2.47 (D-A) | Hydrogen Bonds |
|  | 2 | 38A | ALA | 1.75 (H-A), 2.61 (D-A) | Hydrogen Bonds |
|  | 3 | 40A | VAL | 2.48 (H-A), 3.46 (D-A) | Hydrogen Bonds |
|  | 4 | 41A | ASN | 2.1 (H-A), 3.07 (D-A) | Hydrogen Bonds |
|  | 5 | 69A | ASN | 2.29 (H-A), 3.14 (D-A) | Hydrogen Bonds |
|  | 6 | 70A | GLY | 1.9 (H-A), 2.86 (D-A) | Hydrogen Bonds |
| CAV1_Quercetin | 1 | 64A | VAL | 3.97 | Hydrophobic Interactions |
|  | 2 | 100A | TYR | 3.01 | Hydrophobic Interactions |
|  | 3 | 100A | TYR | 3.76 | Hydrophobic Interactions |
|  | 4 | 127B | ILE | 3.63 | Hydrophobic Interactions |
|  | 1 | 60A | ASN | 2.81 (H-A), 3.75 (D-A) | Hydrogen Bonds |
|  | 2 | 60A | ASN | 2.26 (H-A), 3.07 (D-A) | Hydrogen Bonds |
|  | 3 | 61A | ASP | 3.57 (H-A), 4.06 (D-A) | Hydrogen Bonds |
|  | 4 | 64A | VAL | 1.89 (H-A), 2.72 (D-A) | Hydrogen Bonds |
|  | 5 | 96A | LYS | 2.07 (H-A), 3.05 (D-A) | Hydrogen Bonds |
|  | 6 | 97A | TYR | 2.2 (H-A), 3.18 (D-A) | Hydrogen Bonds |
|  | 7 | 123B | SER | 2.13 (H-A), 3.01 (D-A) | Hydrogen Bonds |
| AR_Kaempferol | 1 | 707A | LEU | 3.77 | Hydrophobic Interactions |
|  | 2 | 745A | MET | 3.33 | Hydrophobic Interactions |
|  | 3 | 749A | MET | 3.97 | Hydrophobic Interactions |
|  | 4 | 873A | LEU | 3.46 | Hydrophobic Interactions |
|  | 1 | 704A | LEU | 1.78 (H-A), 2.53 (D-A) | Hydrogen Bonds |
|  | 2 | 705A | ASN | 2.02 (H-A), 2.68 (D-A) | Hydrogen Bonds |
|  | 3 | 711A | GLN | 3.16 (H-A), 3.54 (D-A) | Hydrogen Bonds |
|  | 4 | 745A | MET | 1.89 (H-A), 2.78 (D-A) | Hydrogen Bonds |
|  | 5 | 749A | MET | 3.7 (H-A), 4.05 (D-A) | Hydrogen Bonds |
|  | 6 | 752A | ARG | 3.38 (H-A), 3.99 (D-A) | Hydrogen Bonds |
|  | 7 | 752A | ARG | 1.87 (H-A), 2.78 (D-A) | Hydrogen Bonds |
|  | 8 | 873A | LEU | 2.09 (H-A), 3.02 (D-A) | Hydrogen Bonds |
|  | 9 | 877A | THR | 3.27 (H-A), 3.81 (D-A) | Hydrogen Bonds |
|  | 10 | 877A | THR | 2.18 (H-A), 2.86 (D-A) | Hydrogen Bonds |
| IGF2_Quercetin | 1 | 44D | GLU | 3.54 | Hydrophobic Interactions |
|  | 2 | 48D | PHE | 3.26 | Hydrophobic Interactions |
|  | 3 | 48D | PHE | 3.51 | Hydrophobic Interactions |
|  | 4 | 49D | ARG | 3.29 | Hydrophobic Interactions |
|  | 1 | 41D | GLY | 2.01 (H-A), 2.67 (D-A) | Hydrogen Bonds |
|  | 2 | 44D | GLU | 2.36 (H-A), 3.03 (D-A) | Hydrogen Bonds |
|  | 3 | 45D | GLU | 1.84 (H-A), 2.8 (D-A) | Hydrogen Bonds |
|  | 4 | 48D | PHE | 2.04 (H-A), 2.8 (D-A) | Hydrogen Bonds |
|  | 5 | 49D | ARG | 2.11 (H-A), 3.03 (D-A) | Hydrogen Bonds |
|  | 6 | 49D | ARG | 3.27 (H-A), 3.97 (D-A) | Hydrogen Bonds |
| ALB_Beta-carotene | 1 | 10A | ARG | 3.96 | Hydrophobic Interactions |
|  | 2 | 14A | LEU | 3.39 | Hydrophobic Interactions |
|  | 3 | 22A | LEU | 3.26 | Hydrophobic Interactions |
|  | 4 | 22A | LEU | 2.99 | Hydrophobic Interactions |
|  | 5 | 26A | ALA | 3.34 | Hydrophobic Interactions |
|  | 6 | 27A | PHE | 3.6 | Hydrophobic Interactions |
|  | 7 | 27A | PHE | 3.72 | Hydrophobic Interactions |
|  | 8 | 46A | VAL | 3.96 | Hydrophobic Interactions |
|  | 9 | 46A | VAL | 3.84 | Hydrophobic Interactions |
|  | 10 | 46A | VAL | 3.03 | Hydrophobic Interactions |
|  | 11 | 49A | PHE | 3.62 | Hydrophobic Interactions |
|  | 12 | 69A | LEU | 3.29 | Hydrophobic Interactions |
|  | 13 | 69A | LEU | 3.43 | Hydrophobic Interactions |
|  | 14 | 70A | PHE | 3.77 | Hydrophobic Interactions |
|  | 15 | 72A | ASP | 3.66 | Hydrophobic Interactions |
|  | 16 | 73A | LYS | 3.53 | Hydrophobic Interactions |
|  | 17 | 76A | THR | 3.88 | Hydrophobic Interactions |
|  | 18 | 151A | ALA | 3.85 | Hydrophobic Interactions |
|  | 19 | 152A | PRO | 3.43 | Hydrophobic Interactions |
|  | 20 | 250A | LEU | 3.14 | Hydrophobic Interactions |
|  | 21 | 251A | LEU | 3.68 | Hydrophobic Interactions |
| CTNNB1_Beta-carotene | 1 | 150A | THR | 3.08 | Hydrophobic Interactions |
|  | 2 | 153A | ILE | 3.47 | Hydrophobic Interactions |
|  | 3 | 154A | PRO | 3.8 | Hydrophobic Interactions |
|  | 4 | 154A | PRO | 3.41 | Hydrophobic Interactions |
|  | 5 | 157A | THR | 3.21 | Hydrophobic Interactions |
|  | 6 | 158A | LYS | 3.5 | Hydrophobic Interactions |
|  | 7 | 161A | ASN | 3.35 | Hydrophobic Interactions |
|  | 8 | 187A | ALA | 3.85 | Hydrophobic Interactions |
|  | 9 | 190A | ARG | 3.12 | Hydrophobic Interactions |
|  | 10 | 193A | GLN | 3.9 | Hydrophobic Interactions |

Index: Coding of the interaction

AA: Amino Acid Type

Distance: Distance between interactions carbon atoms

Distance (H-A): Distance between hydrogen and acceptor atoms

Distance (D-A): Distance between donor and acceptor atoms

**Table S6.** KEGG pathway analysis results of common targets

| **ID** | **Description** | **p-value** | **Genes** |
| --- | --- | --- | --- |
| hsa04010 | MAPK signaling pathway | 5.20E-09 | MET, CHUK, TGFB1, MYC, IGF2, FLT3, ERBB2, CACNA1S, TP53, INSR, RASA1, AKT1, PDGFRA, IL1B, RPS6KA3 |
| hsa05205 | Proteoglycans in cancer | 4.87E-08 | MET, COL1A1, CTNNB1, ESR1, TGFB1, MYC, IGF2, ERBB2, CAV1, TP53, AKT1, PIK3R1 |
| hsa04151 | PI3K-Akt signaling pathway | 1.60E-05 | MET, COL1A1, CHUK, MYC, IGF2, FLT3, ERBB2, TP53, INSR, AKT1, PIK3R1, PDGFRA |
| hsa05215 | Prostate cancer | 3.19E-09 | SRD5A2, CHUK, CTNNB1, ERBB2, RB1, TP53, AKT1, PIK3R1, PDGFRA, AR |
| hsa05163 | Human cytomegalovirus infection | 8.68E-06 | CASP8, CHUK, CTNNB1, MYC, RB1, TP53, AKT1, PIK3R1, PDGFRA, IL1B |
| hsa04080 | Neuroactive ligand-receptor interaction | 0.000469611 | S1PR2, THRB, EDNRA, NR3C1, DRD2, EDNRB, F2, AVPR2, HTR2A, PRSS1 |
| hsa05226 | Gastric cancer | 2.10E-06 | MET, CTNNB1, TGFB1, MYC, ERBB2, RB1, TP53, AKT1, PIK3R1 |
| hsa05160 | Hepatitis C | 3.25E-06 | CASP8, CHUK, CTNNB1, MYC, IFNG, RB1, TP53, AKT1, PIK3R1 |
| hsa05225 | Hepatocellular carcinoma | 5.67E-06 | MET, CTNNB1, TGFB1, MYC, IGF2, RB1, TP53, AKT1, PIK3R1 |
| hsa05417 | Lipid and atherosclerosis | 4.10E-05 | CASP8, PPARG, CHUK, MMP1, TP53, AKT1, PIK3R1, IL1B, CD40LG |
| hsa04014 | Ras signaling pathway | 8.20E-05 | MET, CHUK, IGF2, FLT3, INSR, RASA1, AKT1, PIK3R1, PDGFRA |
| hsa05230 | Central carbon metabolism in cancer | 5.96E-08 | MET, MYC, FLT3, ERBB2, TP53, AKT1, PIK3R1, PDGFRA |
| hsa05418 | Fluid shear stress and atherosclerosis | 1.16E-05 | CHUK, CTNNB1, IFNG, CAV1, TP53, AKT1, PIK3R1, IL1B |
| hsa05224 | Breast cancer | 1.75E-05 | CTNNB1, ESR1, MYC, ERBB2, RB1, TP53, AKT1, PIK3R1 |
| hsa05161 | Hepatitis B | 3.54E-05 | CASP8, CHUK, TGFB1, MYC, RB1, TP53, AKT1, PIK3R1 |
| hsa05167 | Kaposi sarcoma-associated herpesvirus infection | 0.000126477 | CASP8, CHUK, CTNNB1, MYC, RB1, TP53, AKT1, PIK3R1 |
| hsa04510 | Focal adhesion | 0.000161686 | MET, COL1A1, CTNNB1, ERBB2, CAV1, AKT1, PIK3R1, PDGFRA |
| hsa05415 | Diabetic cardiomyopathy | 0.000173113 | COL1A1, CTSD, TGFB1, COL3A1, SLC2A4, INSR, AKT1, PIK3R1 |
| hsa05207 | Chemical carcinogenesis - receptor activation | 0.000233112 | ESR1, MYC, CACNA1S, RB1, AKT1, PIK3R1, AR, RPS6KA3 |
| hsa05166 | Human T-cell leukemia virus 1 infection | 0.000318843 | CHUK, TGFB1, MYC, CHEK2, RB1, TP53, AKT1, PIK3R1 |
| hsa05212 | Pancreatic cancer | 1.89E-06 | CHUK, TGFB1, ERBB2, RB1, TP53, AKT1, PIK3R1 |
| hsa05220 | Chronic myeloid leukemia | 1.89E-06 | CHUK, TGFB1, MYC, RB1, TP53, AKT1, PIK3R1 |
| hsa05142 | Chagas disease | 1.36E-05 | CASP8, CHUK, TGFB1, IFNG, AKT1, PIK3R1, IL1B |
| hsa04919 | Thyroid hormone signaling pathway | 4.13E-05 | THRB, CTNNB1, ESR1, MYC, TP53, AKT1, PIK3R1 |
| hsa04380 | Osteoclast differentiation | 5.92E-05 | PPARG, CHUK, TGFB1, IFNG, AKT1, PIK3R1, IL1B |
| hsa04926 | Relaxin signaling pathway | 6.23E-05 | COL1A1, EDNRB, TGFB1, MMP1, COL3A1, AKT1, PIK3R1 |
| hsa04936 | Alcoholic liver disease | 0.000114299 | ACOX1, CASP8, CHUK, CTNNB1, ACADM, AKT1, IL1B |
| hsa04932 | Non-alcoholic fatty liver disease | 0.000197327 | CASP8, PPARG, TGFB1, INSR, AKT1, PIK3R1, IL1B |
| hsa04218 | Cellular senescence | 0.000205332 | TGFB1, MYC, CHEK2, RB1, TP53, AKT1, PIK3R1 |
| hsa05164 | Influenza A | 0.000360401 | CASP8, CHUK, IFNG, AKT1, PIK3R1, IL1B, PRSS1 |
| hsa05202 | Transcriptional misregulation in cancer | 0.000745134 | MET, PPARG, MPO, MYC, FLT3, RUNX2, TP53 |
| hsa05169 | Epstein-Barr virus infection | 0.000974844 | CASP8, CHUK, MYC, RB1, TP53, AKT1, PIK3R1 |
| hsa04015 | Rap1 signaling pathway | 0.001223006 | MET, DRD2, CTNNB1, INSR, AKT1, PIK3R1, PDGFRA |
| hsa04024 | cAMP signaling pathway | 0.001642521 | ACOX1, EDNRA, DRD2, CACNA1S, PDE10A, AKT1, PIK3R1 |
| hsa04020 | Calcium signaling pathway | 0.002623757 | MET, EDNRA, EDNRB, ERBB2, CACNA1S, PDGFRA, HTR2A |
| hsa05213 | Endometrial cancer | 5.45E-06 | CTNNB1, MYC, ERBB2, TP53, AKT1, PIK3R1 |
| hsa00140 | Steroid hormone biosynthesis | 7.34E-06 | HSD11B2, SRD5A2, CYP19A1, CYP11B1, CYP17A1, STS |
| hsa05221 | Acute myeloid leukemia | 1.27E-05 | MPO, CHUK, MYC, FLT3, AKT1, PIK3R1 |
| hsa05218 | Melanoma | 1.93E-05 | MET, RB1, TP53, AKT1, PIK3R1, PDGFRA |
| hsa05223 | Non-small cell lung cancer | 1.93E-05 | MET, ERBB2, RB1, TP53, AKT1, PIK3R1 |
| hsa05210 | Colorectal cancer | 5.33E-05 | CTNNB1, TGFB1, MYC, TP53, AKT1, PIK3R1 |
| hsa05222 | Small cell lung cancer | 7.80E-05 | CHUK, MYC, RB1, TP53, AKT1, PIK3R1 |
| hsa01522 | Endocrine resistance | 0.000110963 | ESR1, ERBB2, RB1, TP53, AKT1, PIK3R1 |
| hsa04933 | AGE-RAGE signaling pathway in diabetic complications | 0.000124136 | COL1A1, TGFB1, COL3A1, AKT1, PIK3R1, IL1B |
| hsa05146 | Amoebiasis | 0.000138521 | COL1A1, TGFB1, COL3A1, IFNG, PIK3R1, IL1B |
| hsa05145 | Toxoplasmosis | 0.00023153 | CASP8, CHUK, TGFB1, IFNG, AKT1, CD40LG |
| hsa04068 | FoxO signaling pathway | 0.000538602 | CHUK, TGFB1, SLC2A4, INSR, AKT1, PIK3R1 |
| hsa04210 | Apoptosis | 0.000656914 | CASP8, CHUK, CTSD, TP53, AKT1, PIK3R1 |
| hsa05162 | Measles | 0.000737023 | CASP8, CHUK, TP53, AKT1, PIK3R1, IL1B |
| hsa04072 | Phospholipase D signaling pathway | 0.001023429 | F2, AVPR2, INSR, AKT1, PIK3R1, PDGFRA |
| hsa05152 | Tuberculosis | 0.002776484 | CASP8, CTSD, TGFB1, IFNG, AKT1, IL1B |
| hsa05219 | Bladder cancer | 1.54E-05 | MYC, MMP1, ERBB2, RB1, TP53 |
| hsa05144 | Malaria | 4.12E-05 | MET, TGFB1, IFNG, IL1B, CD40LG |
| hsa01524 | Platinum drug resistance | 0.000254354 | CASP8, ERBB2, TP53, AKT1, PIK3R1 |
| hsa05214 | Glioma | 0.000288769 | RB1, TP53, AKT1, PIK3R1, PDGFRA |
| hsa01521 | EGFR tyrosine kinase inhibitor resistance | 0.000368101 | MET, ERBB2, AKT1, PIK3R1, PDGFRA |
| hsa04211 | Longevity regulating pathway | 0.000638332 | PPARG, TP53, INSR, AKT1, PIK3R1 |
| hsa04657 | IL-17 signaling pathway | 0.000819139 | CASP8, CHUK, MMP1, IFNG, IL1B |
| hsa04620 | Toll-like receptor signaling pathway | 0.001292296 | CASP8, CHUK, AKT1, PIK3R1, IL1B |
| hsa04625 | C-type lectin receptor signaling pathway | 0.001292296 | CASP8, CHUK, AKT1, PIK3R1, IL1B |
| hsa04660 | T cell receptor signaling pathway | 0.001292296 | CHUK, IFNG, AKT1, PIK3R1, CD40LG |
| hsa04931 | Insulin resistance | 0.001529224 | SLC2A4, INSR, AKT1, PIK3R1, RPS6KA3 |
| hsa04066 | HIF-1 signaling pathway | 0.001593148 | ERBB2, IFNG, INSR, AKT1, PIK3R1 |
| hsa04668 | TNF signaling pathway | 0.001796752 | CASP8, CHUK, AKT1, PIK3R1, IL1B |
| hsa04071 | Sphingolipid signaling pathway | 0.002345479 | S1PR2, CTSD, TP53, AKT1, PIK3R1 |
| hsa04152 | AMPK signaling pathway | 0.002432819 | PPARG, SLC2A4, INSR, AKT1, PIK3R1 |
| hsa04611 | Platelet activation | 0.002806024 | COL1A1, F2, COL3A1, AKT1, PIK3R1 |
| hsa04110 | Cell cycle | 0.003007448 | TGFB1, MYC, CHEK2, RB1, TP53 |
| hsa05216 | Thyroid cancer | 0.000188403 | PPARG, CTNNB1, MYC, TP53 |
| hsa04213 | Longevity regulating pathway - multiple species | 0.001374076 | SOD1, INSR, AKT1, PIK3R1 |
| hsa05211 | Renal cell carcinoma | 0.002043907 | MET, TGFB1, AKT1, PIK3R1 |
| hsa04917 | Prolactin signaling pathway | 0.002155132 | ESR1, CYP17A1, AKT1, PIK3R1 |
| hsa04520 | Adherens junction | 0.002270474 | MET, CTNNB1, ERBB2, INSR |
| hsa03320 | PPAR signaling pathway | 0.002774544 | ACOX1, PPARG, MMP1, ACADM |
| hsa05100 | Bacterial invasion of epithelial cells | 0.00305326 | MET, CTNNB1, CAV1, PIK3R1 |
| hsa04960 | Aldosterone-regulated sodium reabsorption | 0.0029707 | HSD11B2, INSR, PIK3R1 |


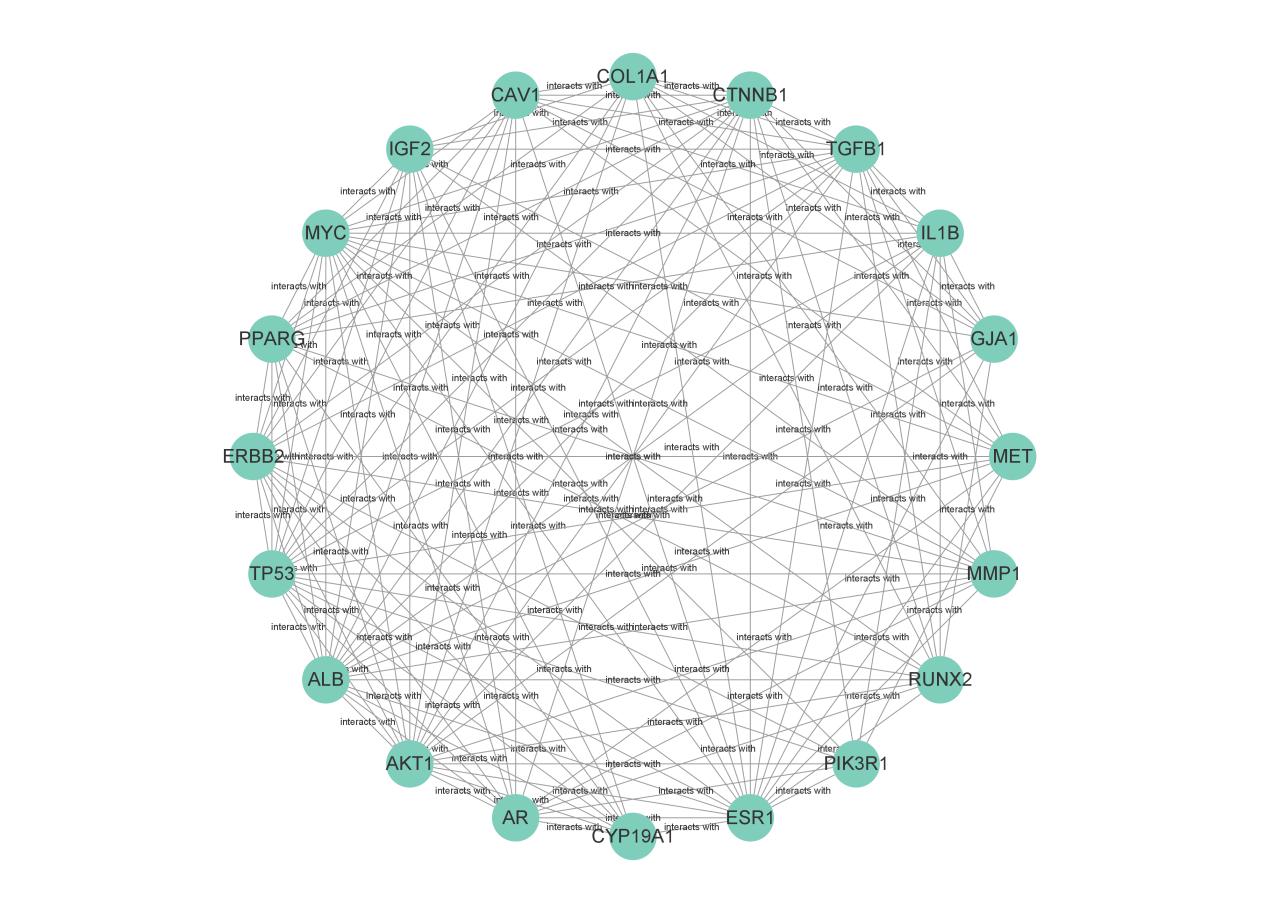


FIGURE S1: The protein complex or functional module in PPI network of common targets.


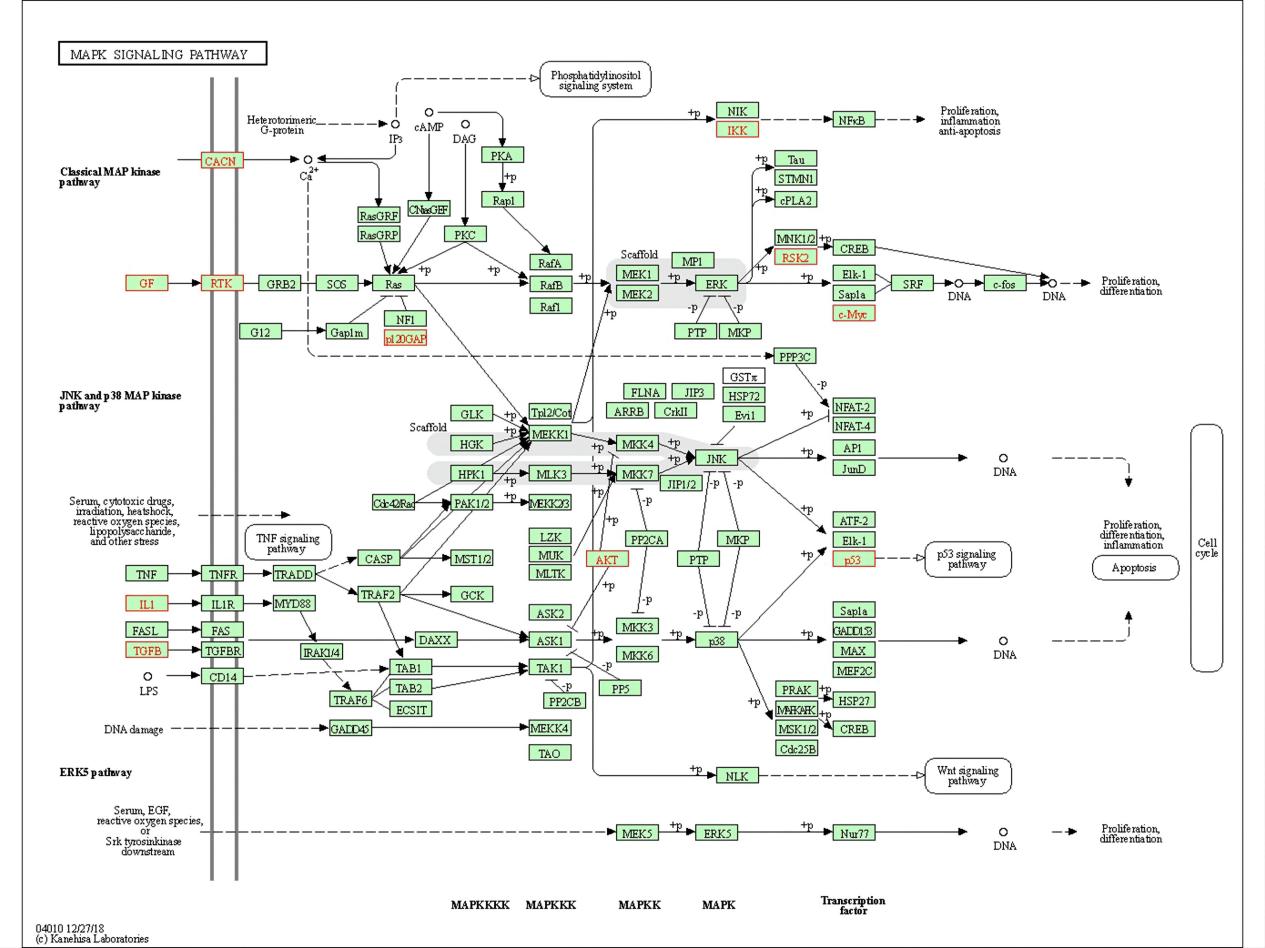


FIGURE S2: The MAPK signaling pathway. Genes marked in red indicate targets of DJD involved in this pathway.


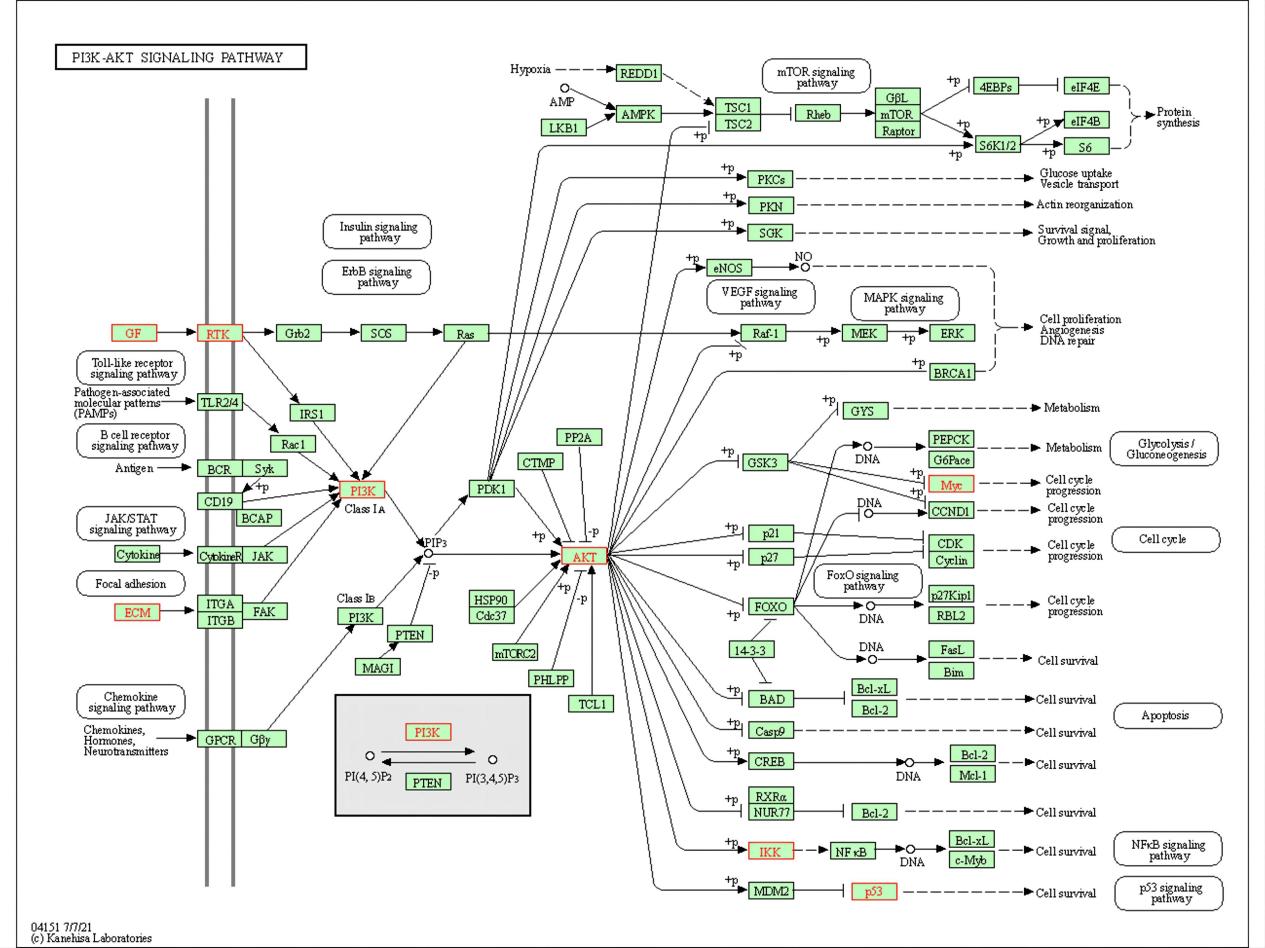


FIGURE S3: The PI3K/AKT signaling pathway. Genes marked in red indicate targets of DJD involved in this pathway.
