# Supplementary figures and images for: A Study Based on Network Pharmacology Decoding the Multi-Target Mechanism of Duhuo Jisheng Decoction for the Treatment of Intervertebral Disc Degeneration
Source: Comput Intell Neurosci. 2023 May 28;2023:7091407. doi: 10.1155/2023/7091407 (PMC10243954; doi:10.1155/2023/7091407)

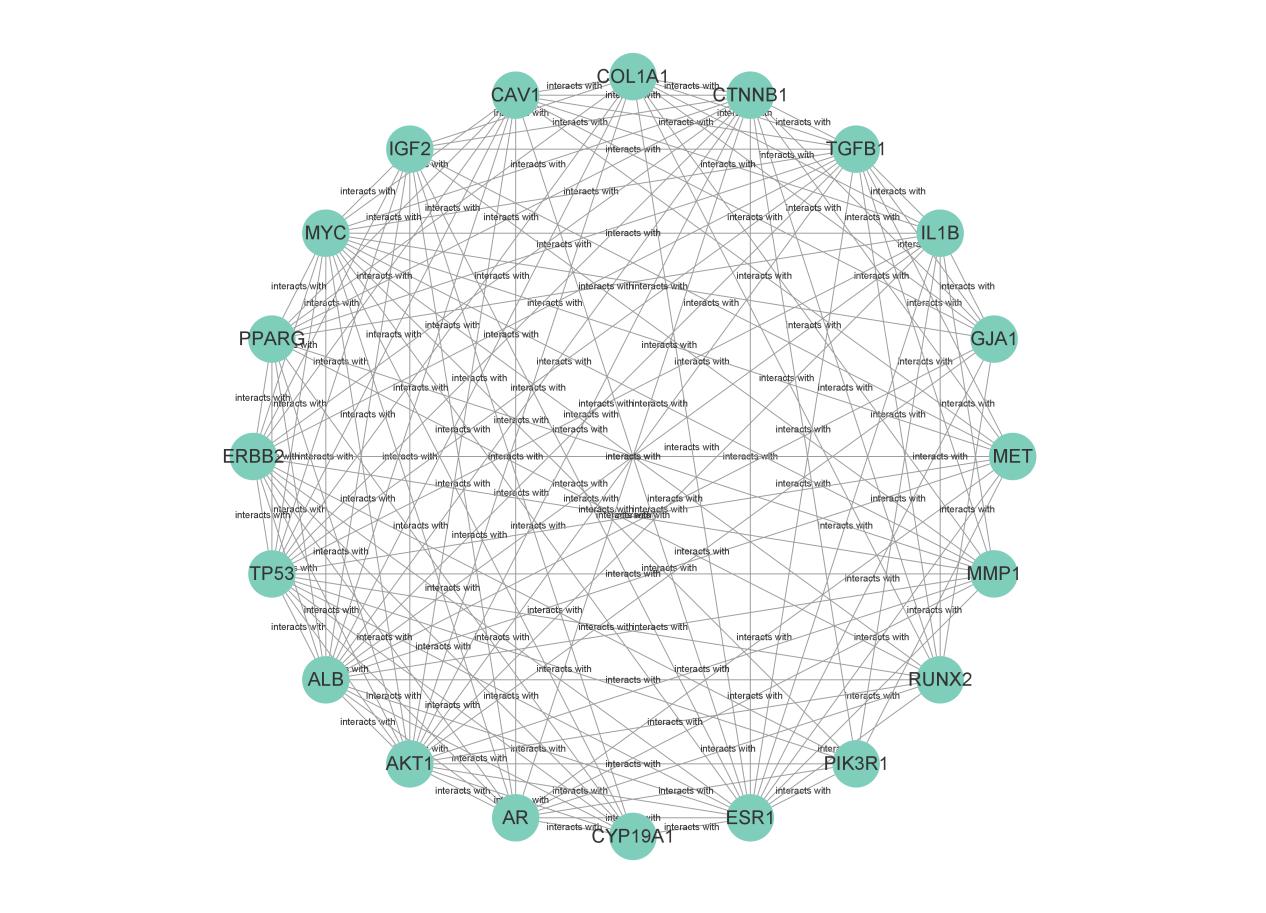

Supplement: Supplementary Materials — Table S1 records Lipinski's rule of five (RO5). Table S2 details the renaming results of the compounds in DJD. Table S3 shows the information on receptor proteins and docking sites. Table S4 lists the 67 DJD compounds that we screened for oral bioavailability and therapeutic potential. Table S5 details the protein-ligand interaction information. Table S6 exhibits the results of KEGG pathway analysis of common targets. Figure S1 shows the protein complex or functional module in the PPI network of common targets. Figure S2 shows the target genes of DJD involved in MAPK signaling pathway. Figure S3 shows the target genes of DJD involved in PI3K/AKT signaling pathway. [file 7091407.f1.zip › Figure. S1 (1).jpg]

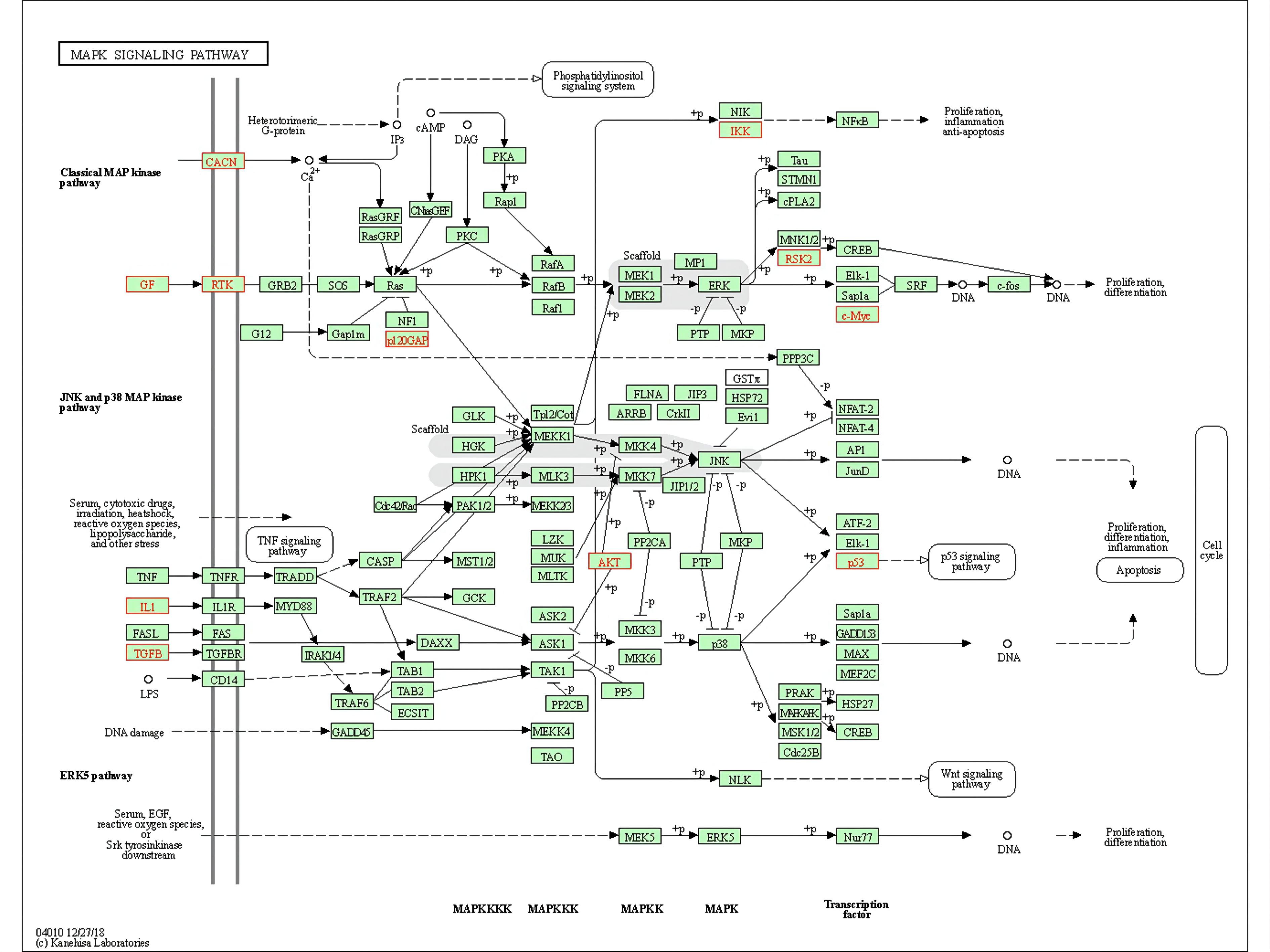

Supplement: Supplementary Materials — Table S1 records Lipinski's rule of five (RO5). Table S2 details the renaming results of the compounds in DJD. Table S3 shows the information on receptor proteins and docking sites. Table S4 lists the 67 DJD compounds that we screened for oral bioavailability and therapeutic potential. Table S5 details the protein-ligand interaction information. Table S6 exhibits the results of KEGG pathway analysis of common targets. Figure S1 shows the protein complex or functional module in the PPI network of common targets. Figure S2 shows the target genes of DJD involved in MAPK signaling pathway. Figure S3 shows the target genes of DJD involved in PI3K/AKT signaling pathway. [file 7091407.f1.zip › Figure. S2 (1).jpg]

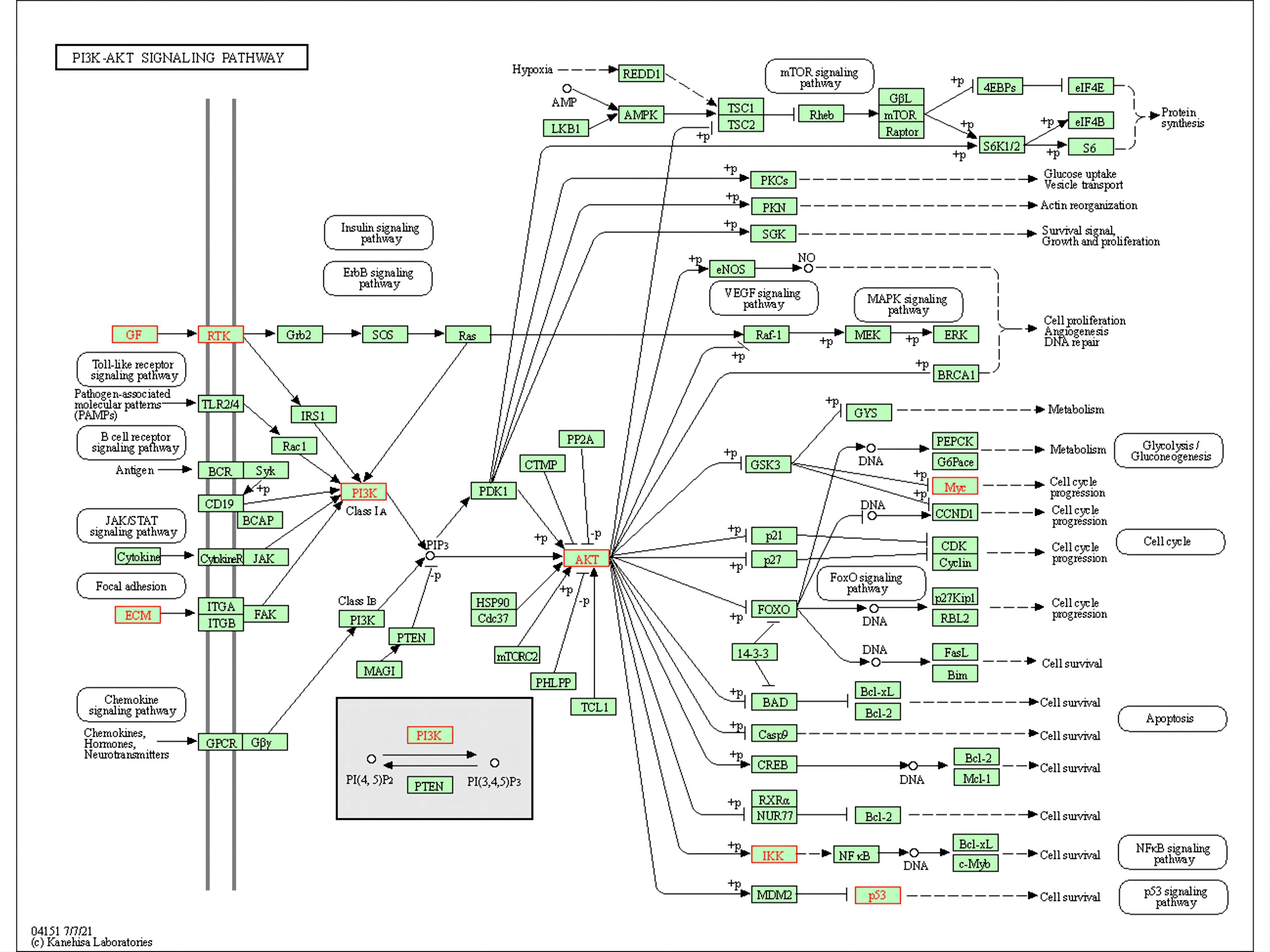

Supplement: Supplementary Materials — Table S1 records Lipinski's rule of five (RO5). Table S2 details the renaming results of the compounds in DJD. Table S3 shows the information on receptor proteins and docking sites. Table S4 lists the 67 DJD compounds that we screened for oral bioavailability and therapeutic potential. Table S5 details the protein-ligand interaction information. Table S6 exhibits the results of KEGG pathway analysis of common targets. Figure S1 shows the protein complex or functional module in the PPI network of common targets. Figure S2 shows the target genes of DJD involved in MAPK signaling pathway. Figure S3 shows the target genes of DJD involved in PI3K/AKT signaling pathway. [file 7091407.f1.zip › Figure. S3 (1).jpg]
